# A close look at current γδ T-cell immunotherapy

**DOI:** 10.3389/fimmu.2023.1140623

**Published:** 2023-03-31

**Authors:** Ling Ma, Yanmin Feng, Zishan Zhou

**Affiliations:** ^1^ Institute of Molecular Medicine, College of Future Technology, Peking University, Beijing, China; ^2^ Research and Development Department, Beijing Dingchengtaiyuan (DCTY) Biotech Co., Ltd., Beijing, China

**Keywords:** γδ T cells, adoptive cell transfer (ACT), cancer immunotherapy, clinical trial, immunotherapy

## Abstract

Owing to their antitumor and major histocompatibility complex (MHC)-independent capacities, γδ T cells have gained popularity in adoptive T-cell immunotherapy in recent years. However, many unknowns still exist regarding γδ T cells, and few clinical data have been collected. Therefore, this review aims to describe all the main features of the applications of γδ T cells and provide a systematic view of current γδ T-cell immunotherapy. Specifically, this review will focus on how γδ T cells performed in treating cancers in clinics, on the γδ T-cell clinical trials that have been conducted to date, and the role of γδ T cells in the pharmaceutical industry.

## 1 Introduction

There are two types of T cells: αβ T cells and γδ T cells. The former expresses a T-cell receptor (TCR) comprising a heterodimer of α and β chains. The latter expresses a TCR comprising a heterodimer of γ and δ chains, which normally do not express the co-receptors CD4 and CD8, and account for, on average, 4% of human peripheral blood T cells (1). γδ T cells as a whole link the innate and adaptive immune responses. However, when referring to γδ T cells, it should be noted that they are not a homogeneous population, but rather a heterogeneous group of cells with diverse properties (**Table 1**) (2). Based on the TCR δ chain variable gene expression, human γδ T cells are normally divided into Vδ2 T cells and non-Vδ2 T cells, with Vδ1- and Vδ3-expressing γδ T cells accounting for the majority of non-Vδ2 T cells (2, 3). The distribution and frequency of γδ T cell subset differ dramatically in tissues and blood (2). Recently, non-Vγ9Vδ2 T cells have been considered a more appropriate division of γδ T cells and are the dominant γδ T cells in organs and lymphoid tissues, such as the skin, intestine, lungs, liver, lymph nodes, thymus, etc., representing the adaptive-like γδ T cells (2, 4). In human blood, the majority (i.e., approximately 50%–95%) of γδ T cells express a Vδ2 chain paired with a Vγ9 chain (5). This subset (i.e., Vγ9Vδ2 T cells) specifically and universally (*via* semi-invariant polyclonal expansion) recognizes phosphoantigens (PAgs) derived from microbes or transformed cells (6, 7) through butyrophilin (BTN) family members BTN2A1 and BTN3A1 (8–10), representing innate-like γδ T cells.

**Table 1:** Main features of human γδ T cells.

Subset	Distribution	T cells (%)	Ligands	Innate-like receptors	Cytokines
Non-Vγ9Vδ2	Organs and lymphoid tissues (skin, intestine, lung, liver, thymus, lamina propria, decidua, breast, spleen, etc.)	Approximately 0.5–16	BTNL3/BTNL8, CD1a, CD1c, CD1d, MICA, ULBP4, EPCR, HLA-B*5802, β2-microglobulin-free HLA heavy chain, EphA2, MSH2, hsp60, histidyl-tRNA synthetases, PE	NKG2D, TLR, CD16	IFNγ, TNFα, IL-17, IL-4, IL-10, TGFβ
Vγ9Vδ2	Blood	Approximately 2–4	BTN2A1/BTN3A1, F1-ATPase		

BTN, butyrophilin; MICA, MHC class I chain-related protein A; ULBP, UL16-binding protein; EPCR, endothelial protein C receptor; EphA2, ephrin type-A receptor 2; MSH2, MutS homolog 2; hsp60, heat shock protein 60; PE, phycoerythrin.

αβ T cells recognize peptides, lipids, and metabolites presented by the major histocompatibility complex (MHC), CD1, and MHC class I-related protein (MR1), respectively. In contrast, the antigens and ligands recognized by γδ T cells remain largely unknown. Those that have been identified are difficult to classify into clear-cut categories (11–13). Human γδ T cells are MHC independent and have been found to recognize a wide range of ligand molecules, such as BTN family proteins (BTN2A1/BTN3A1 and BTNL3/BTNL8), MHC-related proteins [CD1a, CD1c, CD1d, MHC class I polypeptide-related sequence A (MICA), UL16-binding protein (ULBP4), endothelial protein C receptor (EPCR), HLA-B*5802, and β2-microglobulin-free HLA heavy chain], ephrin type-A receptor 2 (EphA2), and those lacking typical membrane structural proteins [MutS homolog 2 (MSH2), heat shock protein 60 (hsp60), histidyl-tRNA synthetases, phycoerythrin (PE), and mitochondrial F1-ATPase] (12–20). γδ T cells recognize these molecules through their TCRs and also express innate receptors, such as natural killer (NK) receptors (e.g., NKG2D), Toll-like receptors (TLRs), and Fc receptors (e.g., CD16), which recognize ligands such as MICA, MHC class I chain-related (MIC) protein A (MICA), MICB, and UL16-binding protein (ULBP) (21–23).

γδ T cells play a role in fighting infectious and tumorous diseases, as well as a role in homeostasis, wound healing, and aging (4, 19, 24, 25). In mouse studies, γδ T cells also regulate body temperature and shape neurons (2, 26). The immune response of γδ T cells is intrinsically biased toward type I immunity, which exerts strong cytotoxic (mainly through granzyme B and perforin) effects on infected and tumor cells, and results in increased IFNγ production (27). However, the differences in TCR genes between humans and mice (2, 28), especially the absence of Vγ9Vδ2 T cells in common non-primate experimental animals (29, 30), limits the relevance of preclinical *in vivo* studies using mouse models for human Vγ9Vδ2 T cells. Therefore, this review will focus solely on human γδ T cells.

## 2 γδ T cells in cancer

### 2.1 Correlation with clinical prognosis

The relationship between γδ T cells and cancer prognosis is influenced by factors such as the pathological type of cancer (31), the γδ T-cell subset (32), the time of sample harvesting (33), and the functioning state of the γδ T cells (34). Clinical prognosis studies typically involve the analysis of either peripheral γδ T cells or tumor-infiltrating γδ T cells, and common methods include flow cytometry (35), immunohistochemistry (31, 36), and gene expression measurement (34, 37). Early studies often measured peripheral γδ T cells without distinguishing subsets (38), whereas later studies began to analyze subsets separately (35).

Overall, γδ T cells are positively correlated with favorable prognosis in cancerous diseases (39). The earliest observations that suggested that γδ T cells play a positive role in cancer prognosis came from a follow-up study of allogeneic stem cell transplantation for treating acute leukemia in the 1990s (40). Long-term follow-up found 5-year disease-free survival (DFS) and overall survival (OS) rates of 54.4% and 70.8%, respectively, among patients with increased levels of peripheral γδ T cells, compared with 19.1% and 19.6%, respectively, among those without increased γδ T cells, with no difference in graft-versus-host disease rate (38). Later studies of acute leukemia in children also supported this finding (41). Children with a higher percentage of CD8+ γδ T cells, even when sampled before treatment, had a better prognosis (33). Further studies indicate that the Vδ8, Vδ4, and Vγ9 subsets are positively correlated with good prognosis in acute leukemia before treatment (42, 43). However, studies carried out in patients with chronic lymphocytic leukemia found that peripheral Vγ9Vδ2 T-cell numbers before treatment were negatively correlated with disease progression and that those γδ T cells were dysfunctional towards zoledronate stimulation (44). This was also true in patients with chronic myeloid leukemia (45).

In the case of solid tumors, peripheral Vγ9Vδ2 T cells have been found to be positively correlated with OS or progression-free survival (PFS) in patients with renal cell carcinoma (46), melanoma (32, 47), and bladder cancer (37), as determined by flow cytometry. However, the presence of the Vδ1 subset in blood was not found to be favorable in melanoma and bladder cancer. As most studies on solid tumors have focused on tumor-infiltrating cells, the majority of correlations between prognosis and γδ T cells have been found in tumor-infiltrating cells. Effector Vδ1 γδ T cells have been found to be beneficial in skin cancers (32, 48), colon cancer (34), and lung cancer (35), based on protein-level analysis. Similarly, using the more commonly used gene expression analysis, tumor-infiltrating γδ T cells were found to be favorable in ovarian cancer (49), head and neck cancer (50), and bladder cancer (37).

However, the roles of γδ T cells have been found to vary in different pathological types of breast cancer and in different studies. In immunohistochemical studies, Ma et al. found that γδ T cells were negative indicators in non-triple-negative breast cancers (36), whereas Allaoui et al. found the opposite correlation for non-triple-negative cancers, and also found no clear correlations between γδ T-cell infiltration and triple-negative breast cancer (31). It is worth noting that, according to the limited description of their method, Allaoui et al. were comparing the presence and absence of γδ T-cell infiltration, whereas Ma et al. were comparing infiltration with lower and higher numbers of γδ T-cells, which may account for the difference in the findings of the two studies. In other studies, γδ T cells, especially the Vδ1 subset (51), have been more frequently found to predict good outcomes in triple-negative breast cancer that supported by protein-level or gene-level analyses (51, 52). Gene expression analysis, using public databases, indicated that γδ T cells were positively correlated with good outcomes for all types of breast cancer (53, 54). Interestingly, one study found that certain peripheral TCR-γ motifs were positively correlated with OS in breast cancer (55).

In pancreatic cancer, high CD31 levels and low CD73 levels in cancer cells have been found to be associated with increased OS and an increased number of antitumor immune cells, including γδ T cells (56, 57).

### 2.2 Anti-tumor and pro-tumor effects

The immune responses of γδ T cells toward tumor cells have been well summarized in other reviews (5, 27, 58–61). Generally, human γδ T cells are activated when tumor cells bind to their TCRs and/or innate receptors, such as NK cell receptors, in tumor conditions. They exhibit direct cytotoxicity against different types of cancer cells, modulate antitumor cytokines, and interact with other immune cells to eliminate tumors (27), which is in accordance with the favorable prognosis linked to γδ T cells clinically observed in malignant diseases, as reviewed above. Fighting against tumor growth is among the primary roles of human γδ T cells, whether they are peripheral or tissue-resident. However, clinical investigations have also indicated the importance of the functional state of γδ T cells in cancers (32, 35, 44). In tumor environments, γδ T cells can exhibit protumor effects by producing IL-17, recruiting protumor myeloid immune cells, or suppressing αβ T-cell antitumor activities (62–64). The tumor environment tends to educate γδ T cells to serve it and selects the protumor subsets (65). These “conditioned” protumor findings may contribute to the unfavorable prognosis linked to γδ T cells observed in clinics.

## 3 γδ T-cell immunotherapy in cancer

The historical development of adoptive T-cell therapy was discovered through hematopoietic stem cell transplantation (HSCT) and the graft-versus-leukemia effect. This effect showed that patients with graft-versus-host disease had a lower relapse rate and that the depletion of T cells led to a higher relapse rate (66). In the case of γδ T cells, their antitumor properties were observed in the late 1980s *in vitro* (67), but it was not until the early 2000s that Hayday’s group established their antitumor role in mice (27). Soon after that, γδ T cells began to be tested for treating malignant diseases in humans (68).

Knowing the biological features of immune cells is crucial for using them to help fight diseases. As mentioned above, Vγ9Vδ2 T cells are the dominant and most studied subset in human peripheral blood. Their semi-invariant TCRs recognize small non-peptide pyrophosphate antigens through conformational changes after BTN2A1 and BTN3A1 heterodimers bind PAgs intracellularly (8, 9). Typical natural PAgs include isopentenyl pyrophosphate (IPP) and (E)-4-hydroxy-3-methyl-but-2-enyl pyrophosphate (HMBPP), with the latter being the most potent natural PAg currently known (69). PAgs are crucial metabolites that come from the universally present isoprenoid biosynthesis pathway. IPP exists in all organisms, whereas HMBPP is absent in mammals (69). Studies have shown that the level of IPP is increased in abnormal human cells (7). Other than this direct activating mode, aminobisphosphonates (N-BPs) and certain alkylamines can indirectly activate Vγ9Vδ2 T cells through TCRs. They inhibit the downstream enzyme farnesyl pyrophosphate synthase (FPS, or FPPS), which plays a role in IPP synthesis and leads to endogenous IPP accumulation (2, 70). Once activated, the innate-like Vγ9Vδ2 T cells intrinsically differentiate into cytotoxic and antitumor cytokine-producing effector cells. Compared with other γδ T-cell subsets, Vγ9Vδ2 T cells are relatively harvest and to expand *in vitro*. As more is known about non-Vγ9Vδ2 T cells, and the *in vitro* culturing of Vδ1 γδ T cells on a comparable scale has become possible, Vδ1 γδ T cells have started to attract attention and be introduced to γδ T cell immunotherapy (71).

In this section, we will review the strategies used for applying γδ T cells in patients, the results of completed trials of γδ T-cell therapies, and the results to date of ongoing clinical trials of γδ T-cell therapies.

### 3.1 Current strategies in practice

**Figure 1:**
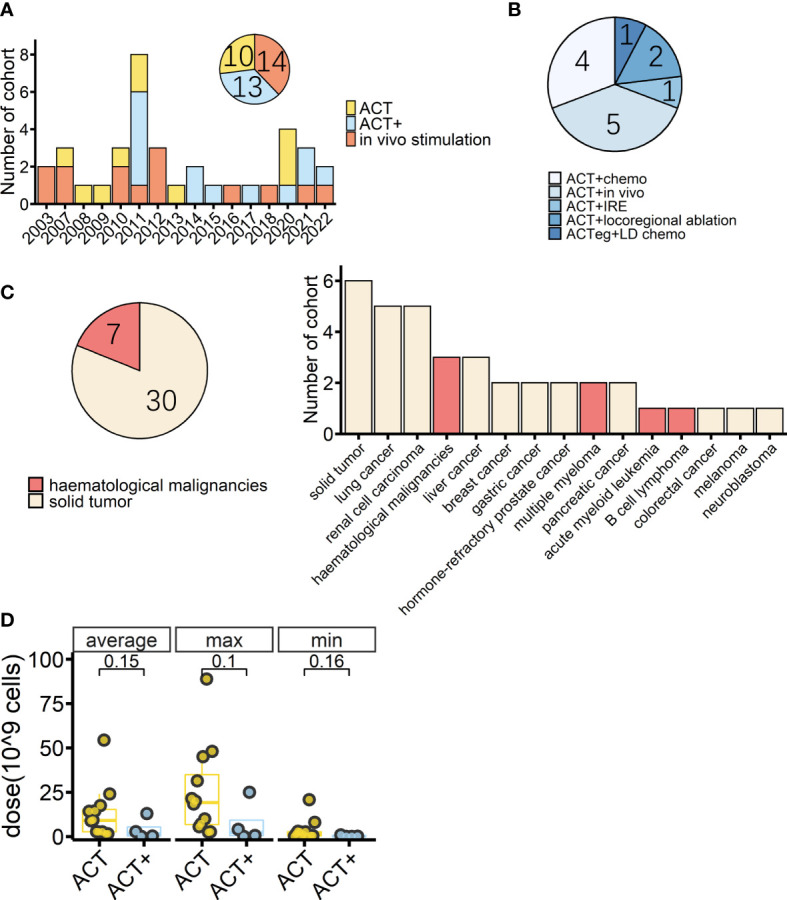
Overview of cohorts from completed γδ T-cell immunotherapy trials. **(A)** Timeline of completed γδ T-cell immunotherapy trials in clinics by cohort; colors indicate treatment type (*n* = 37). **(B)** Detailed treatment methods applied in the cohorts treated with adoptive cell transfer (ACT) combined with other treatments except for IL-2 treatment (ACT+) (*n* = 13). Chemo: conventional therapies, mostly chemotherapy*. In vivo*: *in vivo* stimulation of Vγ9Vδ2 T cells using zoledronate. IRE: irreversible electroporation. ACTeg: engineered T cells. LD chemo: lymphodepletion chemotherapy. **(C)** Tumor types (pie chart) and detailed diseases (bar chart) of γδ T-cell immunotherapy cohorts (*n* = 37); colors indicate treated tumor types. **(D)** Average, maximum, and minimum γδ T-cell total infusion doses of cohorts with dosage information (ACT, *n* = 12, ACT+, *n* = 4). The lower and upper hinges of the boxplot show the 25th and 75th percentile, respectively. The medians are indicated inside the box, and *p*-values (Wilcoxon rank-sum test) are also indicated.

Cancer immunotherapy is about how to safely unleash the anticancer power of immune cells. The first and most important step is to efficiently activate immune cells. Until now, most efforts to apply γδ T cells in clinics have focused on boosting the Vγ9Vδ2 subset because its stimulant effect on the immune system is relatively clear. As mentioned earlier, Vγ9Vδ2 T cells can be directly or indirectly activated by PAgs or N-BPs. N-BPs are conveniently well-established drugs in clinics that are used to treat bone diseases such as osteoporosis, metastatic bone disease, multiple myeloma, and hypercalcemia of malignancy (72). N-BP drugs, such as pamidronate and zoledronate, are usually given intravenously, and, therefore, might readily activate the peripherally dominant Vγ9Vδ2 T cells *in vivo*. Early γδ T-cell trials followed this approach (**Figure 1A**) (68, 73). However, the response rate of patients’ Vγ9Vδ2 T cells toward N-BP drugs was not satisfactory and repeated administrations led to a reduction in peripheral Vγ9Vδ2 T cells. All of these shortcomings would greatly hamper the treatment (68, 74).

Activating Vγ9Vδ2 T cells *in vitro* followed by adoptive transfer to patients would largely avoid these problems. Indeed, shortly after the *in vivo* attempt, researchers started to activate (by either direct stimulants, such as IPP and synthetic PAgs, or indirect stimulants such as zoledronate) the patient’s own Vγ9Vδ2 T cells *in vitro* and then reinfuse the patient with them (75–77). This approach follows the common practice in adoptive T-cell immunotherapy, which uses autologous cells. However, it also shares flaws with current adoptive T-cell immunotherapy, such as the fact that the immune environment in patients often works against antitumor effects and large differences between patients in γδ T-cell numbers and in their capacity to increase the number of γδ T-cells (32, 47, 78, 79). The unique features of γδ T cells also provide a solution to these problems. By taking advantage of their MHC-independent nature (e.g. they can be used in situations where MHC matching between the donor and recipient is not possible; the innate-like recognition of stress-induced antigens (7) provides a fast reaction; and the more limited repertoire of γδ TCRs (80) means that they are less likely to recognize and attack host tissues), the allogeneic adoptive transfer becomes possible. Early HSCT therapies found that using αβ T-cell depleted method to treat patients caused fewer cases of graft-versus-host disease and, importantly, those who developed graft-versus-host disease but had high levels of γδ T cells were less likely to experience relapse (38, 81). These results are encouraging, supporting the use of the allogeneic adoptive transfer of γδ T cells in cancer immunotherapy.

Repeated infusion of allogeneic Vγ9Vδ2 T cells is safe and has shown promising effects in treating liver and lung cancers (82). In recent years, the use of a humanized anti-BTN3A monoclonal antibody (ICT01) to activate Vγ9Vδ2 T cells has been explored as a potential treatment for multiple cancers (83). Preliminary results showed that the treatment was well tolerated, and disease control rates of 22%–42% were achieved in the completed phase I trial (Imchecktherapeutics.com: Imcheck Therapeutics). In addition to the previously mentioned strategies aimed at utilizing the inherent anticancer capabilities of Vγ9Vδ2 T cells, there have been recent human trials investigating γδ T-cell-related T-cell modifications. These modification techniques include the addition of a chimeric antigen receptor (CAR) to γδ T cells to create CAR-γδ T cells; the transfer of antitumor γδ TCR to αβ T cells (TEG001, GDT002, GDT201); the fusion of an antibody anti-CD19 Fab region and the transmembrane andendo domains of a γδ TCR, as well as a separate CD19 single-chain variable fragment (scFv) and a 4-1BB costimulatory molecule, to create a novel T cell (ET019003) to treat B-cell lymphoma (84).

As for the new subject of research, Vδ1 γδ T cells, there are currently two phase I clinical trials in progress. Both trials are using allogeneic Vδ1 T cells. The first trial (NCT05001451) is using non-modified Vδ1 T cells (GDX012) to treat acute myeloid leukemia, while the other trial (NCT04735471) is using anti-CD20 CAR-modified Vδ1 T cells (ADI-001) to treat B-cell malignancies.

### 3.2 Current γδ T-cell immunotherapy clinical results

A PubMed^®^ (National Library of Medicine, Bethesda, MD, USA) search found that, as of October 2022, there were at least 28 studies (37 cohorts) of γδ T-cell immunotherapy in progress in clinics, all of which were using Vγ9Vδ2 T cells. These studies included a total of 559 patients, 396 of whom tumor responses were measurable. As reviewed above, there are different strategies for applying γδ T-cell treatment; thus, in this systematic analysis, *in vivo* stimulation, adoptive cell transfer (ACT, including autologous and allogeneic), and ACT combined with other treatments, except for IL-2 treatment (ACT+), are compared. There were 14 cohorts receiving *in vivo* stimulation and 23 cohorts receiving reinfusion (**Figure 1A**). Early studies were normally of a single treatment, either *in vivo* stimulation or γδ T-cell infusion, with or without IL-2 treatment and vitamin D supply (68, 73, 75–77, 85–87), while later studies started to combine γδ T-cell therapy with other traditional treatments, typically chemotherapy (**Figures 1A, B**). A combination of ACT and *in vivo* stimulation produced the first reported complete response in a metastatic renal cell carcinoma cohort of patients (**Table 2**) (88).

**Table 2:** Current γδ T cell therapy clinical results.

Study	Year	Disease	Age (years) (median)	Treatment type	Treatment	Outcome	Reference
Wilhelm et al.	2003	Lymphoma, multiple myeloma	67.5	*In vivo* stimulation	Pamidronate + IL-2 (continuous IV from d3)	1 SD (10%)	(68)
		Lymphoma, multiple myeloma	52	*In vivo* stimulation	Pamidronate + IL-2 (bolus IV from d1)	3 PR (33%), 2 SD (22%)	
Dieli et al.	2007	Prostate cancer	68	*In vivo* stimulation	Zoledronate + Ca/vitamin D	1 SD (11%), 1 PR (11%)	(73)
		Prostate cancer	68	*In vivo* stimulation	Zoledronate + IL-2 + Ca/vitamin D	2 PR (22%), 4 SD (44%)	
Kobayashi et al.	2007	Renal cell carcinoma	51	ACT	Autologous, IPP-expanded Vγ9Vδ2 T cells + IL-2	3 prolonged tumor doubling time (60%)	(75)
Bennouna et al.	2008	Renal cell carcinoma	57	ACT	Innacell γδ™ + IL-2	6 SD (60%)	(76)
Abe et al.	2009	Multiple myeloma	57.5	ACT	Autologous, zoledronate-expanded Vγ9Vδ2 T cells	4 SD (67%)	(77)
Bennouna et al.	2010	Solid tumor	56	*In vivo* stimulation	BrHPP + IL-2	12 SD (43%)	(85)
Meraviglia et al.	2010	Breast cancer	63	*In vivo* stimulation	Zoledronate + IL-2	1 PR (10%), 2 SD (20%)	(86)
Nakajima et al.	2010	Lung cancer	66	ACT	Autologous zoledronate-expanded Vγ9Vδ2 T cells	3 SD (30%)	(87)
Kobayashi et al.	2011	Renal cell carcinoma	61	ACT+	Autologous IPP-expanded Vγ9Vδ2 T cells + zoledronate + IL-2	1 CR (9%), 5 SD (45%)	(88)
Lang et al.	2011	Renal cell carcinoma	57	*In vivo* stimulation	zoledronate + IL-2	2 SD (17%)	(74)
Nicol et al.	2011	Solid tumor	59.5	ACT+	Autologous zoledronate-expanded Vγ9Vδ2 T cells + zoledronate	2 SD (33%)	(89)
		Solid tumor	61	ACT+	Autologous, zoledronate-expanded Vγ9Vδ2 T cells + zoledronate	1 SD (11%)	
		Solid tumor	44	ACT+	Autologous zoledronate-expanded Vγ9Vδ2 T cells + zoledronate + conventional treatment	1 CR (33%), 2 PR (67%)	
Sakamoto et al.	2011	Lung cancer	67	ACT	Autologous zoledronate-expanded Vγ9Vδ2 T cells	6 SD (40%)	(90)
Noguchi et al.	2011	Solid tumor	60	ACT	Autologous zoledronate-expanded Vγ9Vδ2 T cells	2 SD (50%)	(91)
		Solid tumor	60	ACT+	Autologous zoledronate-expanded Vγ9Vδ2 T cells + zoledronate or conventional therapies	3 PR (30%), 1 SD (10%)	
Kunzmann et al.	2012	Renal cell carcinoma	61	*In vivo* stimulation	Zoledronate + IL-2	3 SD (43%)	(92)
		Melanoma	43.5	*In vivo* stimulation	Zoledronate + IL-2	1 SD (17%)	
		Leukemia	72	*In vivo* stimulation	Zoledronate + IL-2	2 PR (25%), 2 SD (25%)	
Izumi et al.	2013	Colorectal cancer	unclear	ACT	Autologous zoledronate-expanded Vγ9Vδ2 T cells	–	(93)
Wilhelm et al.	2014	Hematological malignancies	67	ACT+	Lymphodepletion + zoledronate + CD4/8 T cell-depleted PBMC	3 CR (75%)	(94)
Wada et al.	2014	Gastric cancer	58	ACT+	Autologous zoledronate-expanded Vγ9Vδ2 T cells + zoledronate	Tumor cells in ascites reduced	(95)
Cui et al.	2015	Gastric cancer	58.5	ACT+	Autologous NK cells, γδ T cells, and/or cytokine-induced killer cells + chemotherapy	PFS: 14 vs 8.5 months (chemo only) in stage III cancers	(96)
Pressey et al.	2016	Neuroblastoma	13.5	*In vivo* stimulation	Zoledronate + IL-2	1 SD (25%)	(97)
Aoki et al.	2017	Pancreatic cancers	65	ACT+	Autologous zoledronate-expanded Vγ9Vδ2 T cells + chemotherapy	Increased γδ T cell percentage in non-recurrent patients	(98)
Sugie et al.	2018	Breast cancer	65	*In vivo* stimulation	Zoledronate + letrozole	OR rate by MRI: 38.2%; by caliper: 50%; by ultrasound: 51.7%;	(99)
Xu et al.	2020	Lung cancer	59.5	ACT	Allogeneic zoledronate/vitamin C-expanded Vγ9Vδ2 T cells	1 SD (10%)	(82)
		Liver cancer	47.5	ACT	Allogeneic zoledronate/vitamin C-expanded Vγ9Vδ2 T cells	1 CR (12.5%), 1 SD (12.5%)	
Lin et al.	2020	Pancreatic cancer	63	ACT+	Allogeneic zoledronate-expanded Vγ9Vδ2 T cells + irreversible electroporation (IRE)	OS: 14.5 vs. 11 months (IRE only); PFS: 11 vs. 8.5 months (IRE only)	(100)
Kakimi et al.	2020	Lung cancer	66	ACT	Autologous zoledronate-expanded Vγ9Vδ2 T cells + IL-2	Median PFS: 95 days; median OS: 418 days; 1 PR (4%), 16 SD (64%)	(101)
Fazzi et al.	2021	Multiple myeloma	60	*In vivo* stimulation	Zoledronate + IL-2 + Ca/vitamin D	8 CR (18%)	(102)
Zhang et al.	2021	Liver cancer	53	ACT+	Allogeneic zoledronate/vitamin C expanded Vγ9Vδ2 T cells + locoregional therapy (LT)	Median OS: 13 vs. 8 months (LT only); median distant PFS: 8 vs. 4 months (LT only)	(103)
		Intrahepatic cholangiocarcinoma	56	ACT+	Allogeneic zoledronate/vitamin C expanded Vγ9Vδ2 T cells + locoregional therapy	Median OS: 9 vs. 8 months (LT only); median distant PFS: 8 vs. 4 months (LT only)	
Zheng et al.	2022	Lung cancer	59	*In vivo* stimulation	Zoledronate + anti-PD-1	PFS: 5.4 vs. 2.8 months (without zoledronate); OS: 16.7 vs. 12.8 months (without zoledronate); 23 PR (44.2%), 16 SD (30.8%)	(104)
He et al.	2022	Lymphoma	52	ACT engineered	ET019003 T cells	6 CR (50%), 4 PR (33.3%), 1 SD (8.3%)	(84)

SD, stable disease; PR, partial response; CR, complete response; OS, overall survival; PFS, progression-free survival.

Overall, 81% of the cohorts received γδ T-cell therapy for the treatment of solid cancers (**Figure 1C**). Patients in these cohorts had a wide range of solid malignancies, with lung cancer and renal cell carcinoma being the most tested tumors (**Figure 1C**, right). Excluding one study in which the patients’ median age was unclear (93), the median age ranged from 13.5 years in those receiving neuroblastoma treatment to 68 years for those receiving treatment for prostate cancer (**Table 2**). The highest median age, 72 years, was in a cohort with hematological cancer receiving treatment for acute myeloid leukemia (92). In addition, in the case of adoptive transfer treatment, information on the total infusion dose was available for 16 cohorts. The medians of the average total doses for ACT and ACT+ treatment were 9.1 × 10^9^ cells and 1.6 × 10^9^ cells, respectively. There were no differences between ACT and ACT+ in terms of total doses (**Figure 1D**, Wilcoxon rank-sum test). γδ T-cell therapy was normally applied through multiple infusions, and the range of total infusion dose varied quite widely among patients (from 0.01 × 10^9^ cells to 88.8 × 10^9^ cells).

**Figure 2:**
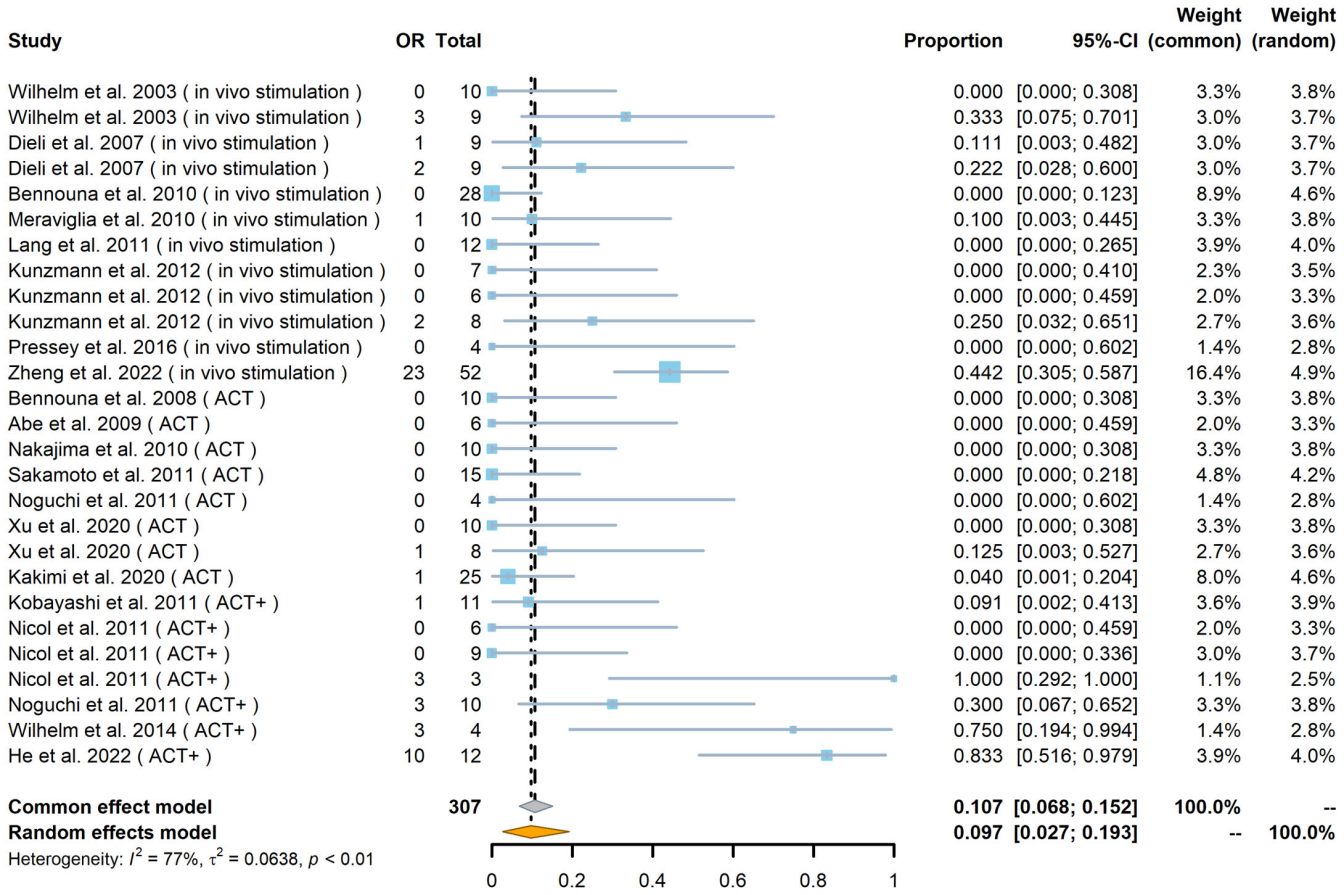
Forest plot of objective response (OR) rate (*n* = 27). The OR indicates the number of patients in each cohort who achieved an objective response. The total indicates the total number of measurable patients in each cohort. The OR rate, 95% confidence interval (CI), and weights of fixed- and random-effects models are indicated for each cohort. Blue squares show the mean OR rate of each cohort, and the size indicates the weight of the cohort; gray lines show the 95% CI, and the diamond shapes show the pooled weighted means of the OR rate using fixed- and random-effects models.

**Figure 3:**
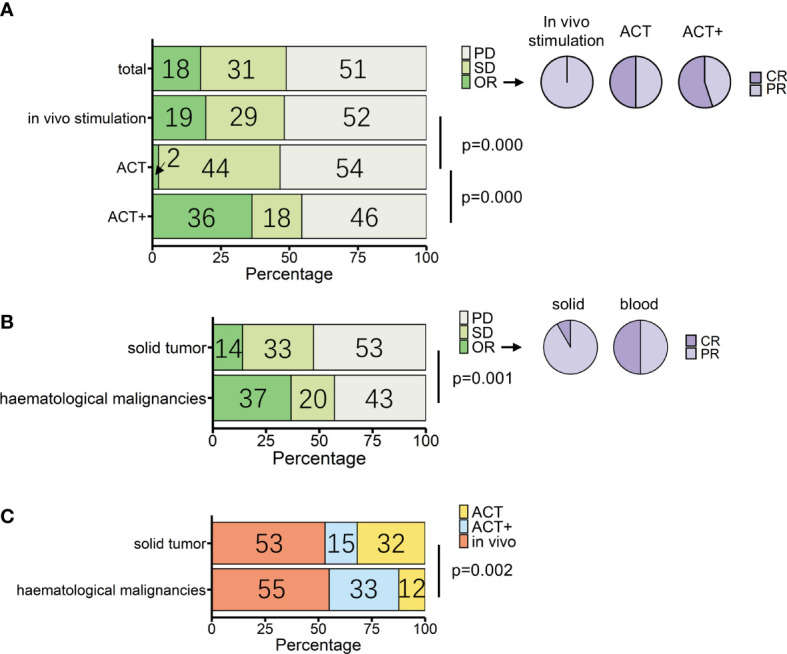
Overview of patients treated with γδ T-cell immunotherapy (*n* = 307). **(A)** Proportions of treatment outcomes in total measurable patients and different treatment types [*in vivo* stimulation, *n* = 164; adoptive cell transfer (ACT), *n* = 88; ACT combined with other treatments except for IL-2 treatment (ACT+), *n* = 55]. Pie charts show the proportions of complete response (CR) and partial response (PR) within objective response (OR) patients for each treatment method (*in vivo* stimulation, *n* = 32; ACT, *n* = 2; ACT+, *n* = 20). **(B)** Proportions of treatment outcomes in different types of tumors (solid, *n* = 258; hematological, *n* = 49). Pie charts show the proportions of CR and PR within OR patients for each tumor type (solid, *n* = 36; hematological, *n* = 18). **(C)** Proportions of treatment types applied in different types of tumors (solid, *n* = 258; hematological, *n* = 49). *p*-values (Pearson’s chi-squared test) are indicated in **(A–C)**. PD, progressive disease; SD, stable disease; OR, objective response; CR, complete response; PR, partial response.

Of the 307 patients (from 27 cohorts) whose tumor responses could be measured (not including the 89 patients whose outcomes were recorded only in terms of OS and PFS), the overall results, regardless of strategy, were an objective response (OR) rate of 18% [including complete response (CR) and partial response (PR), with a pooled OR rate of 9.7%, 95% confidence interval (CI) 2.7%–19.3%; **Figure 2**] and a stable disease (SD) rate of 31% (pooled SD rate of 27.6%, 95% CI 20.3%–35.5%; **Supplemental Figure 1**), based on criteria from RECIST (Response Evaluation Criteria in Solid Tumors) or RECIST v.1.1 (**Figures 2**, **3A**).

The three strategies had statistical differences in outcomes (*p *= 0.000, Pearson’s chi-squared test). According to single-rate meta-analysis, the pooled OR rate for *in vivo* stimulation, ACT, and ACT+ was 8.7% (95% CI 1.1%–20.3%), 0.5% (95% CI 0%–5%), and 35.5% (95% CI 4.8%–73.3%), respectively (**Supplemental Figure 2**).

When compared separately using the number of patients from each condition, *in vivo* stimulation vs. ACT (*p* = 0.000, Pearson’s chi-squared test; **Figure 3A**) and ACT vs. ACT+ (*p* = 0.000, Pearson’s chi-squared test; **Figure 3A**) showed significant differences, while *in vivo* stimulation vs. ACT+ (*p* = 0.03, Pearson’s chi-squared test) were similar at a significance level of 0.017. However, when examining the results in detail, it was found that the best response to *in vivo* stimulation treatments was only PR, with the largest contribution coming from the study that combined *in vivo* stimulation with PD-1 treatment (104). In contrast, 11 out of the 20 OR patients treated with ACT+ achieved CR (**Figure 3A**, pie chart). Furthermore, as tumor type can have a significant impact on treatment response, the responses of patients with hematological cancers and solid cancers were compared in this group of 307 patients (**Figure 3B**, **Supplemental Figure 4**). Of these, 49 patients had hematological cancer, whereas 258 patients had solid cancers. Among hematological cancer patients, 37% (18 patients) achieved OR (pooled OR rate 30.1%, 95% CI 3.2%–65.7%), and among solid tumor patients 14% (36 patients) achieved OR (pooled OR rate 5.6%, 95% CI 0.7%–13.1%).

The two cancer types had significantly different responses to γδ T-cell therapy in general (*p* = 0.001, Pearson’s chi-squared test). However, conclusions should be drawn with caution, as the above analysis shows that the choice of treatment strategy also greatly influences patient response. Among patients with a measurable response, the choice of treatment strategy differed between hematological cancers and solid tumors (*p* = 0.002, Pearson’s chi-squared test), with 32% of solid cancer patients being treated with the lower response rate ACT treatment and 33% of blood cancer patients being treated with ACT+ treatment (**Figure 3C**, **Table 3**). It was not possible to compare the responses of different treatments separately in patients with hematological cancers and those with solid tumors, as only the number of patients with solid tumors was sufficient for statistical analysis. In patients with solid tumors, as in patients overall, there were differences in response between treatments (*p* = 0.003, Pearson’s chi-squared test; **Table 3**), with *in vivo* stimulation and ACT+ showing better OR results than ACT alone [OR rates of 19.7% for *in vivo* stimulation and 17.9% for ACT+ vs. 2.4% for ACT alone; pooled OR rates for *in vivo* stimulation, ACT+, and ACT alone of 6.9% (95% CI 0.0%–20.2%), 18.4% (95% CI 0.0%–58.3%), and 0.6% (95% CI 0.0%–5.5%), respectively, **Supplemental Figure 6**].

**Table 3:** Treatment strategy influences in γδ T cell immunotherapy (*n* = 307).

Treatment differences between hematological and solid tumors				Pearson’s chi-squared test
	*In vivo*	ACT	ACT+	*p* = 0.002
Hematological	27	6	16	
Solid	137	82	39	
Hematological tumors				
	OR	SD	PD	NA
*In vivo*	5	5	17	
ACT	0	4	2	
ACT+	13	1	2	
Solid tumors				
	OR	SD	PD	*p* = 0.003
*In vivo*	27	42	68	
ACT	2	35	45	
ACT+	7	9	23	

NA, not applicable for statistical analysis; OR, objective response; PD, progressed disease; SD, stable disease.

**Figure 4:**
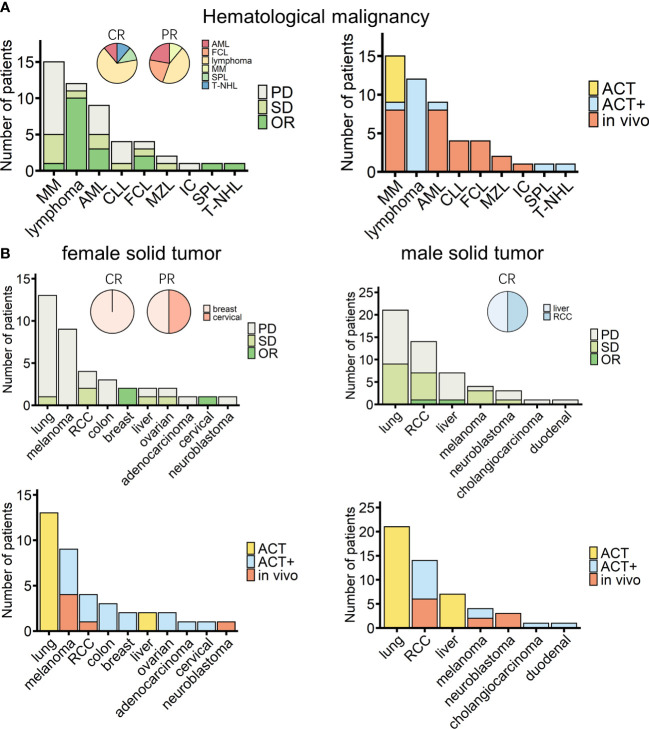
Overview of cancer types of patients treated with γδ T-cell immunotherapy (*n* = 138). **(A)** Diseases (left) and chosen treatment strategies (right) of hematological cancer patients (*n* = 49). Pie charts show the proportions of patients who achieved a complete response (CR) or a partial response (PR) after treatment (CR, *n* = 9; PR, *n* = 9). **(B)** Diseases (upper) and chosen treatment strategies (lower) of female (left) and male (right) solid tumor patients (female, *n* = 38; male, *n* = 51). Pie charts show the proportions of patients who achieved a CR or PR after treatment (female CR, *n* = 1; female PR, *n* = 2; male CR, *n* = 2). MM, multiple myeloma; AML, acute myeloid leukemia; CLL, chronic lymphocytic leukemia; FCL, follicle center lymphoma; MZL, mantle zone lymphoma; IC, immunocytoma; SPL, secondary plasma cell leukemia; T-NHL, T-cell non-Hodgkin lymphoma; RCC, renal cell carcinoma.

Among the 138 patients for whom detailed information, such as age and sex, was recorded alongside treatment responses, no significant differences depending on these characteristics were observed (**Table 4**, **Supplemental Figure 10**). Of these 138 patients, 49 and 89 patients suffered from hematological and solid cancers, respectively. Nine hematological malignancies were treated, with lymphoma being the most tested tumor [including B-cell lymphoma (lymphoma), follicle center lymphoma (FCL), mantle zone lymphoma (MZL), and T-cell non-Hodgkin lymphoma (T-NHL); **Figure 4A**]. A recent cohort study that used modified γδ T cells to treat B-cell lymphoma had the best response rate (**Figure 4A**, **Table 2**) (84). For solid tumors, there were 12 kinds of cancers recorded, with 10 cancers in female patients (*n* = 38) (**Figure 4B**, left panels) and seven cancers in male patients (*n* = 51) (**Figure 4B**, right panels). The type of cancer found most often in tested patients of both sexes was lung cancer, which was treated in all cases with ACT (**Figure 4B**, lower panels); however, the outcomes were moderate (**Figure 4B**, upper panels). Out of 38 female solid cancer patients, three reached OR, all with ACT+ treatment. Although treatment strategy matters, it is interesting to note that, among the ACT+-treated tumors, these positively responding cancers were all female-related (**Figure 4B**, upper left, pie chart). However, the sample size was too small to draw a conclusion. Among male solid cancer patients, 55% were treated with ACT, two patients reached OR, and the only CR patient received ACT+ treatment (**Figure 4B**, right panels).

**Table 4:** Sex, age, tumor type, and treatment strategy influences in γδ T cell immunotherapy (*n* = 138).

Row (R)	Column (C)	Pearson’s chi-squared test (*n* = 138)	Pearson’s chi-squared test (*n* = 46)
Sex	Responses	*p* = 0.214	*p* = 0.444
	Treatments	*p* = 0.619	*p* = 0.122
	Age	*p* = 0.018	*p* = 0.137
	Tumor type	*p* = 0.789	*p* = 0.365
Age	Responses	*p* = 0.087	NA (*p* = 0.005)
	Treatments	*p* = 0.351	*p* = 0.112
	Tumor type	*p* = 1.000	*p* = 0.156
Tumor type	Response	*p* = 0.000	NA (*p* = 0.001)
	Treatment	*p* = 0.000	*p* = 1.000
Treatments	Responses	*p* = 0.000	NA (*p* = 0.003)

Comparisons: sex (female vs. male), age (≤ 60 years vs. > 60 years), tumor type (hematological malignancies vs. solid tumor), treatment [in vivo stimulation, adoptive cell transfer (ACT), and ACT combined with other treatments except for IL-2 treatment (ACT+) and responses [objective response (OR), stable disease (SD), and progressed disease (PD)]. The n = 138 sample comprises all the patients with detailed information corresponding to treatment outcomes. The n = 46 sample comprises patients who received γδ T-cell infusions and with indicated infusion cell numbers. NA, not applicable for statistical analysis.

**Figure 5:**
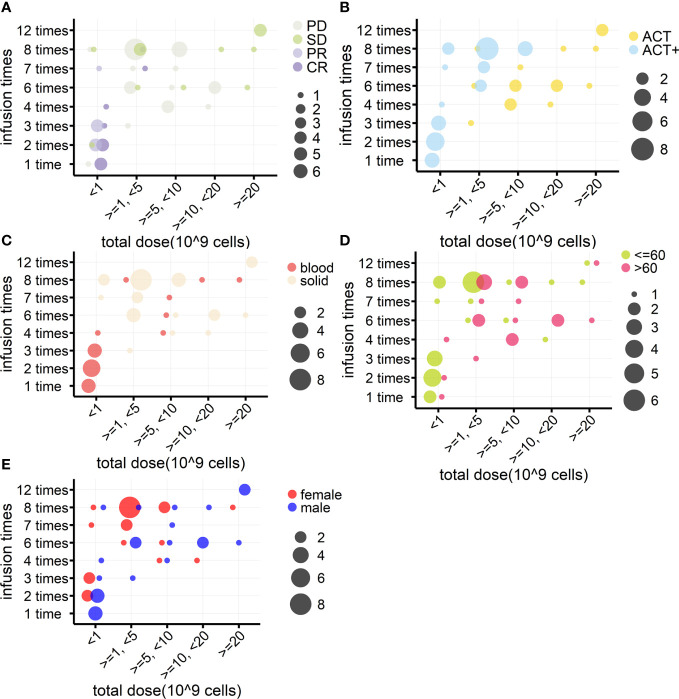
Relationships between γδ T cell reinfusion doses and treatment outcome, strategy, tumor type, age, and sex (*n* = 46). **(A)** Relationship between treatment outcome and dosage. γδ T-cell total infusion cell numbers (10 (9) cells) were divided into five groups and are presented on the *x*-axis; the *y*-axis indicates total infusion times. The bubble color indicates the treatment outcome, and the bubble size indicates the number of patients. **(B)** Relationship between treatment strategy and dosage. **(C)** Relationship between tumor type and dosage. **(D)** Relationship between age and dosage. **(E)** Relationship between sex and dosage.

In addition, when investigating if the number of cells reinfused in patients was associated with outcomes, it was found that, among the 94 patients from eight cohorts treated by γδ T-cell reinfusion, dosage information was available for 46 patients (four cohorts). In general, this sample followed the tendency of the above statistical analysis (**Table 4**, *n* = 46), except that there were no differences in the frequency of use of each strategy between hematological and solid cancers. Although the sample size was not large enough for statistical analysis, the response rates of blood and solid cancers were quite different (**Table 4**, *n* = 46; **Figures 5A–C**). This was mainly due to the cohort with lymphoma that was treated with modified T cells. This cohort also influenced the difference in responses between age groups (**Table 4**, *n* = 46; **Figures 5A–D**), as 75% of the patients in this cohort were less than 60 years old (84), whereas the other three cohorts had equal numbers of patients above and below 60 years old. Overall, the total cell dose tended to be positively correlated with infusion times (**Figure 5**) and ranged from 0.1 × 10^9^ cells to 31.4 × 10^9^ cells. Interestingly, those with an OR response were not necessarily reinfused with the highest γδ T-cell numbers, but all received ACT+ treatment (**Figures 4A, B**, **Table 5**). This was in accordance with the fact that total infusion doses were generally higher in the case of the less effective ACT treatment than when ACT was combined with other treatments (**Figure 5B**, **Table 5**). However, higher doses tended to stabilize disease progression (**Figure 5A**, **Table 5**). When considering only unmodified T-cell therapies (*n* = 34), as the cohort using modified T-cells strongly influence the analysis, the dosages were significantly different when comparing treatment types and sex (**Table 4**). Male patients tended to be infused with higher doses of Vγ9Vδ2 T cells (**Figure 5E**, **Table 5**).

**Figure 6:**
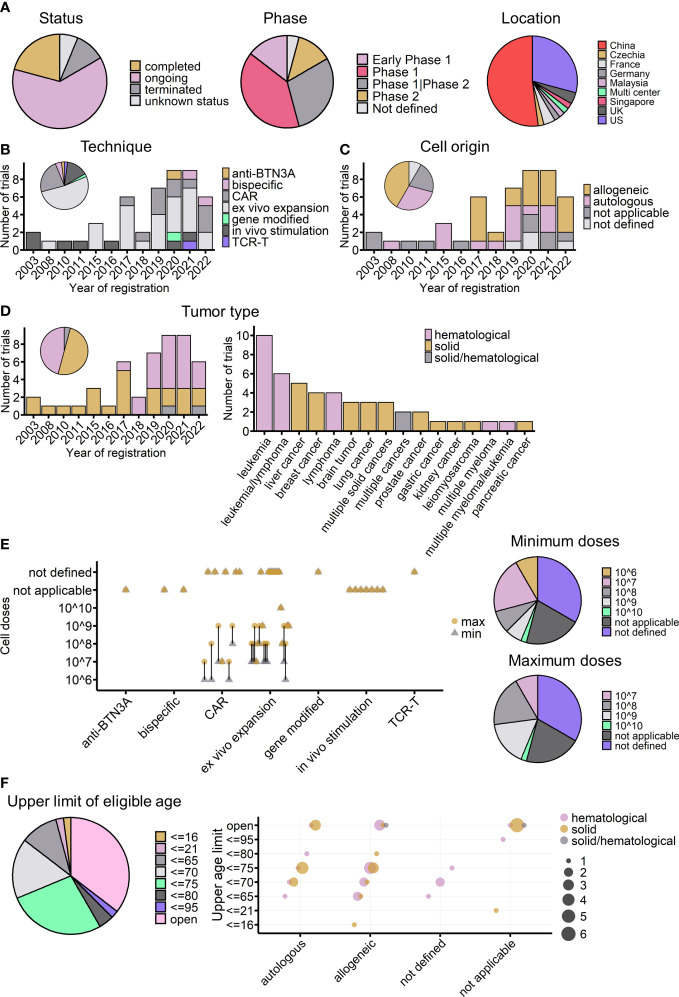
Overview of currently registered γδ T-cell immunotherapy clinical trials (*n* = 48). **(A)** Proportions of currently registered γδ T-cell immunotherapy clinical trials according to status, phase, and location. **(B)** Timeline of registered clinical trials by treatment strategy. The pie chart shows the proportion of trials for each strategy. **(C)** Timeline of registered clinical trials by infusion cell origin. **(D)** Timeline of registered clinical trials by tumor type (left) and detailed malignancies involved in registered trials (right). The pie chart indicates the proportions of different tumor types across all registered trials. **(E)** Planned infusion dosage of registered trials. Left: treatment strategies are indicated on the *x*-axis, and planned γδ T-cell infusion cell numbers are indicated on the *y*-axis. The round yellow dot shows the planned maximum infusion dose, and the gray triangle dot shows the planned minimum infusion dose. The bar connects the maximum and minimum number for each trial. Right: the proportions of different planned minimum (upper) and maximum (lower) infusion doses. **(F)** Upper age limit of registered γδ T-cell immunotherapy clinical trials. Left: proportions of different upper age limits. Right: the relationship between upper age limits, treatment strategy usages, and tumor types. The bubble color shows the tumor type of the trial, and the bubble size indicates the number of trials. **(C–F)** Not applicable: strategies not involving cell infusion; not defined: infusion cell origin not defined.

**Table 5:** Cell dosage differences by treatment outcome, strategy, tumor type, age, and sex in γδ T cell immunotherapy (*n* = 46).

		Median dose (10^9^ cells) (*n* = 46)	*p*-value (Wilcoxon test) (*n = *46)	Median dose (10^9^ cells) (*n = *34)	*p*-value (Wilcoxon test) (*n = *34)
Responses	OR	0.486	OR vs. SD: 0.003; OR vs. PD: 0.001	3.6	ns
	SD	7.2		9.6	
	PD	4.5		4.6	
Treatments	ACT	10.2	0.000	10.2	0.000
	ACT+	0.95		2.5	
Tumor type	Blood	0.52	0.008	7.8	ns
	Solid	3.8		3.8	
Age (years)	≤60	1.25	ns	3.6	ns
	>60	4.7		5.2	
Sex	Female	3	ns	3.5	0.046
	Male	2.6		7.2	

The n = 46 sample comprises patients who received γδ T cell infusions and with indicated infusion cell numbers. The n = 34 sample comprises patients who were reinfused with unmodified γδ T cells. ns, not significant.

### 3.3 Currently registered γδ T cell clinical trials

As of 15 November 2022, at least 48 γδ T cell-related clinical trials had been registered on the ClinicalTrials.gov website. Of these trials, 30 (63%) were ongoing (**Figure 6A**, left), and most of them were phase I trials aimed at testing the safety and preliminary efficacy of using Vγ9Vδ2 T cells to treat cancers (**Figure 6A**, middle). China and the United States accounted for 81% of the initiators of all these trials (**Figure 6A**, right). Compared with the completed trials analyzed above, these registered trials show the dynamic evolution of strategies for applying γδ T cells in clinics (**Figure 6B**). The earliest γδ T-cell clinical trials registered on ClinicalTrials.gov were started in 2003, the same year that Wilhelm et al. published their study on the use of γδ T cells to treat hematological malignancies (68). More than a decade later, the registration rate was quite stable; from 2003 to 2016, only nine γδ T-cell clinical trials were started.

Interestingly, since 2017, there has been a dramatic increase in the number of γδ T-cell clinical trials (**Figures 5B–D**). The first clinical trial using modified γδ T cells (CAR-T) was posted in 2016 and started in 2017 in China, the same year that the FDA (US Food and Drug Administration) approved the world’s first CAR-T cell immunotherapy (Kymriah) to treat leukemia. Since then, 10 more CAR-modified γδ T-cell therapies, one gene-modified study focused on increasing γδ T cells’ chemo drug-resistance (105) (NCT04165941), and one study applying TCR-T cell technology, which transduces tumor cytotoxic γδ TCR to αβ T cells (106, 107) (NCT04688853), have been started. In addition to manipulating T cells directly, the use of Vγ9Vδ2 T-cell-activating antibodies, either humanized anti-BTN3A antibodies (83) (NCT04243499) or engineered tumor-γδ TCR bispecific antibodies (NCT05369000, NCT04887259), has become a new trend in the last 2 years. Antibody drugs have many advantages; for example, they can be obtained “off the shelf”, they are easy to produce, it is easy to ensure quality and to operate batch control, and they are relatively low cost. For therapies involving γδ T-cell infusion, 2017 was also a turning point, with healthy donor-derived γδ T cells arriving on the market. Of the 34 adoptive cell transfer trials after 2017, after excluding four trials that did not indicate cell origin, 20 out of 30 clinical trials used allogeneic γδ T cells (**Figure 6C**). When looking at the treated cancer types, before 2017, γδ T-cell therapy was dedicated to treating solid tumors, but this changed after the launch of CAR-γδ T cell therapy (**Figure 6D**). Currently registered CAR-γδ T-cell therapy trials are mainly testing its use in hematological malignancies, following the successful use of typical CAR-T-cell therapy. Leukemia and liver cancers are the most tested blood and solid cancers, respectively (**Figure 6D**, right).

In the case of adoptive cell transfer, 22 of the trials provided dosage information. There are two ways of calculating dosage: one is based on the body weight of patients, giving the infusion cell number per kg, and the other is based on the total cell number per infusion, regardless of body weight. To easily analyze the two systems together and compare the results, as far as possible, with the results of completed trials the cell number was multiplied by 10 for those trials that considered patients’ weight for dosage. The expected infusion doses were mainly between 10^7^ and 10^8^ cells per infusion (**Figure 6E**). It is important to note that, among the 22 trials, excluding those that did not indicate infusion times, six trials used a single infusion, and eight used multiple infusions (mostly four or six infusions). Thus, when considering body weight and the number of infusions (multiple infusion trials also tended to have a higher dose per infusion), the maximum total planned dose may increase to 10^9^ cells for most trials. The planned doses were similar to those administered to the 16 cohorts in completed trials, in which the median of average cell dose (regardless of ACT or ACT+) was 5.85 × 10^9^ cells (**Figure 1D**), and the medians of the minimum and maximum infusion doses were 0.75 × 10^9^ and 14.15 × 10^9^ cells, respectively.

Furthermore, regarding the T-cell subtype, except for three trials that transferred γδ TCR or part of γδ TCR to αβ T cells, all the other trials used γδ T cells. Currently, only two trials are using allogeneic Vδ1 γδ T cells to treat hematological malignancies (NCT04735471 and NCT05001451); all the other registered clinical trials are focused on the use of Vγ9Vδ2 T cells. As mentioned earlier, the number of Vγ9Vδ2 T cells gradually declines with age (108, 109); this might be considered as a possible influencing factor on treatment outcomes, especially in trials using autologous γδ T cells or trials applying *in vivo* stimulation or activating antibodies. Regarding exclusion criteria, 35% of the registered trials did not set an upper limit on the eligible age of participants (**Figure 6F**), whereas 27% of the trials used an age below 75 years as an inclusion criterion. Among the 14 trials using autologous γδ T cells, 10 trials included either a lower age limit of 75 years or no age limit in their inclusion criteria. The majority of *in vivo* stimulation and Vγ9Vδ2 T-cell-activating antibodies trials do not have an upper age limit (**Figure 6F**). Indeed, for early-phase clinical studies, age limitation was not the priority for exclusion criteria. In future, if possible, the effect of age should be carefully tested and compared during these early phases to give better treatment choices for different age groups of patients. 

## 4 Use of γδ T cells in the pharmaceutical industry

Although many features of γδ T cells are still unknown, their practical uses can still be studied and applied. Currently, at least 29 pharmaceutical companies have made progress in developing γδ T-cell-based therapies for fighting cancers. **Table 6** lists both the preclinical and clinical stage γδ T-cell-related anticancer products being developed by these companies. Compared with the currently registered clinical trials, the approaches designed by the pharmaceutical industry are more diverse.

**Table 6:** Preclinical and clinical stage therapeutic γδ T cells in the pharmaceutical industry.

Company	Drug	Approach	Biological	Condition
Acepodia	ACE1831	Antibody–cell conjugation	Allogeneic, anti-CD20, Vδ2 T cells	Lymphoma
	ACE2016	Antibody–cell conjugation	Allogeneic, anti-EGFR, Vδ2 T cells	EGFR-expressing solid tumors
	ACE1708	Antibody–cell conjugation	Allogeneic, anti-PD-L1, Vδ2 T cells	PD-L1-expressing cancers
	ACE1975	Antibody–cell conjugation	Allogeneic, target undisclosed, Vδ2 T cells	Undisclosed
	ACE2023	Antibody–cell conjugation	Allogeneic, target undisclosed, Vδ2 T cells	Undisclosed
Adicet	ADI-001	CAR	Allogeneic, anti-CD20, Vδ1 T cells	Lymphoma
	ADI-925	Engineered chimeric adapter (CAd)	Allogeneic, tumor stress ligands, Vδ1 T cells	Multiple cancers
	ADI-***	–	Anti-CD70	Multiple cancers
	ADI-***	–	Anti-PSMA	Prostate cancer
	ADI-***	–	Anti-B7-H6	Multiple cancers
	ADI-***	–	Undisclosed	Multiple myeloma
	ADI-***	–	Undisclosed	Solid tumors
	ADI-002	CAR	Allogeneic, anti-GPC3, IL-15 secreting, Vδ1 T cells	Liver cancer
American Gene Technologies	ImmunoTox	Genetic medicine delivered to tumor cells	Up-regulate PAgs in tumor cells to activate Vγ9Vδ2 T cells in situ	Prostate and liver cancer
AVM Biotechnology	AVM0703	High-concentration dexamethasone phosphate drug	–	Lymphoma, leukemia
Beijing Doing Biomedical	NCT02585908s	Unmodified	Autologous, γδ T cells	Gastric cancers
	NCT02656147	CAR	Allogeneic, anti-CD19 CAR γδ T cells	Lymphoma, leukemia
Beijing GD Initiative Cell Therapy Technology	NCT04518774	Unmodified	Allogeneic, γδ T cells	Liver cancer
	NCT04696705	Unmodified	Allogeneic, γδ T cells	Lymphoma
	NCT04028440	Unmodified	Autologous, γδ T cells	Lymphoma, leukemia
Century Therapeutics	CNTY-102	Induced pluripotent stem cells, CAR	Allogeneic, anti-CD19, anti-CD79b, iPSC-derived γδ TCR+, CAR+ T cells CAR-iT)	B-cell malignancies
	CNTY-104	Induced pluripotent stem cells, CAR	Allogeneic, multi-specific, CAR-iT or CAR-iNK	Leukemia
	CNTY-106	Induced pluripotent stem cells, CAR	Allogeneic, multi-specific, CAR-iT or CAR-iNK	Multiple myeloma
	CNTY-***	Induced pluripotent stem cells, CAR	Allogeneic, undisclosed, CAR-iT or CAR-iNK	Solid tumors
CytoMed Therapeutics	CTM-N2D	CAR	Allogeneic, anti-NKG2DL, CAR γδ T cells	Solid tumors
	GDNKT	Induced pluripotent stem cells (iPSC)	Autologous, iPSC-derived γδ NKT cells	Solid tumors
	CTM-GDT	Undisclosed	Allogeneic, γδ T cells	Solid tumors
Eureka(Beijing) Biotechnology	ET190 (ET019003 in NCT04014894, ET019002 in NCT03642496)	Antibody redirected T cells with endogenous modular immune signaling and a co-stimulatory molecule (ARTEMIS^®^)	Autologous, αβ T cells expressing anti-CD19 Fab-γδ TCR intracellular domain and co-stimulatory molecule	Hematological malignancies
Expression Therapeutics	ET206	Undisclosed	γδ T cells	Neuroblastoma
	ET356, ET406	mRNA and novel CAR	CAR γδ T cells	Lymphoma, leukemia
Gadeta	GDT002	Modified CAR or TCR-T	Autologous, Vγ9Vδ2 TCR-expressing αβ T cells	Multiple myeloma, ovarian cancer
	GDT201	Modified CAR or TCR-T	Autologous, non-Vδ2 γδ TCR-expressing αβ T cells	Colorectal cancer
	GDT3nn	Undisclosed	Undisclosed	Solid tumors
GammaDelta Therapeutics Ltd. (Takeda)	GDX012	Unmodified	Allogeneic, non-modified Vδ1 γδ T cells	Leukemia
Adaptate Biotherapeutics (Takeda)	–	Engager antibody	Anti-Vδ1	Undisclosed
Guangdong GD Kongming Biotech	Undisclosed	Undisclosed	Allogeneic, Vγ9Vδ2 T cells	Multiple cancers
Hebei Senlang Biotechnology	Senl_uγδT-123	CAR	Allogeneic, CAR γδ T cells	Undisclosed (possibly AML NCT04796441, NCT05388305)
ImCheck	ICT01	Monoclonal antibody	Anti-BTN3A	Multiple cancers
	ICT03	Monoclonal antibody	Anti-BTN2A	Multiple cancers
	ICT04–08	Monoclonal antibody	Anti-5 BTNs	Multiple cancers
Immatics (with Editas medicine)	ACTallo^®^	CAR or TCR-T, CRISPR gene editing	Allogeneic, CAR, or TCR-T engineered Vγ9Vδ2 T cells	Undisclosed
IN8bio	INB-200	Gene modification	Autologous, gene-modified chemo drug-resistant γδ T cells	Brain tumor
	INB-100	Unmodified	Allogeneic, γδ T cells	Leukemia
	INB-400	Gene modification	Allogeneic, gene-modified chemo drug-resistant γδ T cells	Glioblastoma
	INB-300	Gene modification, CAR	Gene-modified chemo drug-resistant anti chlorotoxin CAR-expressing γδ T cells	Solid tumors
Kiromic BioPharma	Deltacel™	Unmodified	Allogeneic, γδ T cells	Undisclosed
	Procel™	CAR	Allogeneic, anti-PD-L1 CAR γδ T cells	Undisclosed
	ALEXIS-PRO-1 Procel™	CAR	Allogeneic, anti-PD-L1 CAR γδ T cells	Undisclosed
	Isocel™	CAR	Allogeneic, anti-mesothelin CAR γδ T cells	Undisclosed
	ALEXIS- ISO-1 Isocel™	CAR	Allogeneic, anti-mesothelin CAR γδ T cells	Undisclosed
LAVA Therapeutics	LAVA-051	Bispecific γδ T-cell engaging antibody	Anti-CD1d, anti-γδ TCR	Multiple myeloma, leukemia
	LAVA-1207	Bispecific γδ T-cell engaging antibody	Anti-PSMA, anti-γδ TCR	Prostate cancer
	LAVA-1223	Bispecific γδ T-cell engaging antibody	Anti-EGFR, anti-γδ TCR	Solid tumors
	LAVA-1266	Bispecific γδ T-cell engaging antibody	Anti-CD123, anti-γδ TCR	Hematological malignancies
	LAVA-1278	Bispecific γδ T-cell engaging antibody	Anti-CD40, anti-γδ TCR	Hematological malignancies
Legend Biotech	Undisclosed	Enhance the persistence of CAR-γδ T cells *in vivo*	Undisclosed	Undisclosed
Leucid bio	T2	CAR	Allogeneic, CAR γδ T cells	Undisclosed
One Chain Immunotherapeutics	OC-3	Unmodified	Allogeneic, Vδ1 T cells	Undisclosed
PersonGen BioTherapeutics (Suzhou)	UCAR-γδ T	CAR	Target undisclosed (anti-CD7 in NCT04702841, anti CD19/CD20 in NCT04700319), CAR γδ T cells	Multiple cancers
PhosphoGam	Undisclosed	Unmodified, purification step free	Allogeneic, Vδ2 T cells	Undisclosed
PureTech	LYT-210	Blocking antibody	Anti-Vδ1	Solid tumors
Shattuck	GADLEN	Bispecific engager antibody	BTN3A1/BTN2A1 extracellular domain heterodimer, anti-tumor specific antigen (e.g., CD19)	Undisclosed
TC BioPharm	OmnImmune^®^	Unmodified	Allogeneic, unmodified γδ T cells	Leukemia
	Undisclosed	CAR	Allogeneic, CAR γδ T cells	Multiple cancers
UNICET biotech	Undisclosed	Antibody, cell therapy, CAR	Undisclosed	Undisclosed

EGFR, epidermal growth factor receptor; iT, immunotherapy; PSMA, prostate-specific membrane antigen; GPC3, glypican-3.

All the information can be found on each company’s website under pipeline web pages or scientific introduction web pages. Those with clinical trial register numbers can also be found on the ClinicalTrials.gov website.*** means product number not indicated.

There are four broad types of strategies being employed in the pharmaceutical industry. The first is the unmodified strategy, in which researchers focus on harnessing the natural capacity of effector γδ T cells and exploiting their MHC-independent nature to take advantage of “off-the-shelf”, safe-to-use, and easy-to-produce benefits. This approach involves utilizing both Vδ2 and Vδ1 γδ T cells, focusing on optimizing the expansion steps, such as the products of GammaDelta Therapeutics Ltd. and PhosphoGam.

Second is the modified strategy. Introducing the classical CAR structure to γδ T cells is the starting point for this strategy, with at least nine companies using the typical CAR T-cell technique. The targets of CAR γδ T cells can be classified into two types: one is antigens highly expressed in tumors, such as GPC3 and mesothelin, and the other is receptors, such as NKG2DL and PD-L1 (**Table 6**). One study targeting the former found that PD-L1-targeting CAR (αβ) T cells had increased cytotoxicity toward high-PD-L1-expressing tumor cells (110). In addition, many companies have designed modified CARs. One strategy, similar to TCR-T-cell techniques, is transplanting the selected antitumor γδ TCR into αβ T cells, such as Gadeta, or using γδ TCR domains to modify CARs, which has been developed by companies such as Eureka Biotechnology (Beijing). The other strategy combines induced pluripotent stem cells (iPSCs) and CAR techniques to produce off-the-shelf CAR γδ T cells, such as those produced by Century Therapeutics and CytoMed Therapeutics. Studies of the former type have shown the feasibility of generating iPSC-derived antitumor CAR γδ T cells (111, 112). In addition to modifying γδ T cells by CAR, gene editing by CRISPR has also been commonly used in recent years by companies such as IN8bio and Immatics.

Third is the antibody-based strategy. ImCheck has used anti-BTN antibodies to stimulate Vγ9Vδ2 T cells *in vivo*; in an early analysis from their phase I/II clinical trial, ICT01 (anti-BTN3A) demonstrated a 36% disease control rate in a 22-patient cohort (Imchecktherapeutics.com: Imcheck Therapeutics). Several companies, such as LAVA Therapeutics and Shattuck, have used bispecific antibodies to activate and endow specificity on γδ T cells. PureTech has used an anti-Vδ1 antibody to block protumor Vδ1 γδ T cells (**Table 6**). Acepodia has combined the antibody-based approach with adoptive cell transfer, in which γδ T cells are chemically modified by tumor-specific antibodies.

Last is the use of chemical drugs and the modification of tumors. American Gene Technologies aims to genetically modify tumor cells to increase their PAgs level and activate Vγ9Vδ2 T cells *in situ.* AVM Biotechnology’s preclinical results show that the antitumor effects of high concentrations of the drug dexamethasone phosphate involve the activation of γδ TCR^+^ NKT cells. This drug can be given alone or as a preconditioning agent before CAR-T-cell treatment (113, 114).

## 5 Discussion

The applications of γδ T cells in cancerous diseases have been carefully reviewed here, but several limitations should be taken into consideration. First, we used PubMed and ClinicalTrials.gov only as the primary search databases. For ongoing trials, clinical trial registration websites in the EU, Japan, China, Australia, and New Zealand were not cross-checked. However, the analyzed trials should provide a proper overview of current developments in γδ T-cell immunotherapy. Second, in the review of current γδ T-cell treatment outcomes, the aim was to obtain a general idea of whether or not the use of different treatment strategies could influence outcomes based on the limited sample numbers and diverse conditions of different studies. For instance, we roughly divided the treatment strategies into three categories (i.e., *in vivo* stimulation, ACT, and ACT+), but recent studies have tried to combine *in vivo* stimulation with other treatments, such as checkpoint inhibiter treatment (**Table 2**) (104). In addition, the modification of T cells was included in the ACT+ group so that the ACT group, as far as possible, included only conventional γδ T-cell infusion. Such comparisons will become more accurate as the use of γδ T-cell therapies increases in clinics in the future.

Despite these limitations, several conclusions can be drawn from reviewing past γδ T-cell immunotherapies. First, although not covered in this review, γδ T-cell immunotherapy is a safe approach in clinics, whether it involves *in vivo* stimulation, reinfusion, or autologous or allogeneic reinfusion. In general, adverse events more severe than grade 2 were not directly related to γδ T-cell treatment and could be adequately controlled. Second, the treatment method appears to have a significant impact on the outcomes. Reinfusion of γδ T cells tended to have a greater potential for complete responses than *in vivo* stimulation (**Figure 3A**). Even for the less effective treatment of ACT alone, one of the two patients who achieved OR had a complete response (**Figure 3A**), and ACT treatment had the highest pooled SD rate (**Supplemental Figures 3** and **7**). This may be related to the patient’s response to zoledronate treatment. Many *in vivo* stimulation studies involved a zoledronate sensitivity test when selecting patients (76, 85, 92). Although patients were considered to be responsive to zoledronate, multiple treatments reduced the reaction (74, 90). This would not happen with multi-reinfusion of active γδ T cells, especially with cells of allogeneic origin. In addition, for reinfusion, allogeneic γδ T cells from healthy donors may be more resilient to the tumor environment.

Furthermore, combining γδ T-cell therapy with other conventional therapies or *in vivo* stimulation showed promising results. Studies have shown the stimulatory immunomodulating effects of radiotherapy (115). The activated tumor microenvironment resulting from such combined therapy may help the reinfused effector γδ T cells to operate effectively inside the tumor. Unlike CAR-T-cell treatment, γδ T-cell therapy often involves multiple infusions. Infusion times and dosages were not necessarily positively related to treatment responses (**Figure 5A**). It is interesting to note that male patients tended to receive higher reinfusion dosages than female patients (**Figure 5E**). In addition, it is interesting to note the regional difference (**Supplemental Figures 8**, **9**). This was probably mainly due to when the γδ T-cell therapy was applied, as early studies tended to use the direct stimulation strategy. Thus, most *in vivo* stimulation results came from European countries, whereas reinfusion studies were more often carried out in Asian countries. In the future, it would be interesting to investigate if different treatments perform the same in different countries.

From the findings of prognosis studies, it is evident that the functional state of γδ T cells plays a critical role in cancer treatment. The difference in prognoses based on the role γδ T cells play in acute and chronic hematological cancers highlights the importance of manipulating the functioning state of γδ T cells in future immunotherapy design. Long-term disease settings seem to culture dysfunctional γδ T cells and select the protumor subset (65). Therefore, reinfusing fully functioning γδ T cells that are resistant to the tumor microenvironment (TME) could efficiently control cancer progression. In addition, in contrast to CAR-T-cell or TCR-T-cell immunotherapies, conventional γδ T-cell immunotherapy protocols usually do not include the lymphodepletion step. However, one study found that certain chemotherapy drugs could activate tumor macrophages and help create an antitumor TME (116), suggesting that optimizing treatment protocols could also help to improve γδ T-cell immunotherapy outcomes. Furthermore, in solid tumor treatments in women, the three (8%) instances of OR were in breast and cervical cancer patients. Future studies could focus on investigating if these cancers are more sensitive than other types of cancer to γδ T-cell therapy. This is especially important as breast and gynecological cancers have the highest incidence and mortality rates among female cancers according to the Global Cancer Observatory [Global Cancer Observatory (iarc.fr)]. Moreover, in addition to the “quality” of γδ T cells, multiple reinfusion of large numbers of natural γδ T cells tended to stabilize disease progression (**Figure 5A**, **Supplemental Figure 3**). With their MHC-independent advantages, unmodified allogeneic γδ T cells could serve as a good treatment option for providing late-stage cancer patients with more time before undergoing further tumor-eliminating treatments. In addition, understanding the distinctions between responding and non-responding patients is crucial for enhancing the effectiveness of γδ T-cell immunotherapy. Whether the differences lie in genetics or microenvironments, this knowledge should enhance the understanding of γδ T cells as well as enable researchers to make informed decisions regarding precision treatment.

## Author contributions

LM collected and analyzed the data, made the figures and tables, and wrote the manuscript. ZZ and YF helped to correct the manuscript and gave valuable advice. All authors contributed to the article and approved the submitted version.

## Supplementary material

Supplementary Figure 1 Forest plot of stable disease (SD) rate (*n* = 27). SD indicates the number of patients in each cohort who achieved stable disease. The total indicates the total number of measurable patients in each cohort. The SD rate, 95% confidence interval (CI), and weights of fixed- and random-effects models are indicated for each cohort. Blue squares show the mean SD rate of each cohort, and the size indicates the weight of the cohort; gray lines show the 95% CI, and the diamond shapes show the pooled weighted means of the SD rate using fixed- and random-effects models.

Supplementary Figure 2 Forest plot of objective response (OR) rate sub-grouped by treatment strategy (*n* = 27). The OR indicates the number of patients in each cohort who achieved an objective response. The total indicates the total number of measurable patients in each cohort. The OR rate, 95% confidence interval (CI), and weights of fixed- and random-effects models are indicated for each cohort. Blue squares show the mean OR rate of each cohort, and the size indicates the weight of the cohort; gray lines show the 95% CI, and the diamond shapes indicate the pooled weighted means of OR rates for each treatment subgroup and for all cohorts using fixed- and random-effects models.

Supplementary Figure 3 Forest plot of stable disease (SD) rate subgrouped by treatment strategy (*n* = 27).

Supplementary Figure 4 Forest plot of objective response (OR) rate subgrouped by tumor type (*n* = 27).

Supplementary Figure 5 Forest plot of stable disease (SD) rate subgrouped by tumor type (*n* = 27).

Supplementary Figure 6 Forest plot of objective response (OR) rate in solid tumor cohorts subgrouped by treatment (*n* = 21).

Supplementary Figure 7 Forest plot of stable disease (SD) rate in solid tumor cohorts subgrouped by treatment (*n* = 21).

Supplementary Figure 8 Forest plot of objective response (OR) rate subgrouped by research location (*n* = 27).

Supplementary Figure 9 Forest plot of stable disease (SD) rate subgrouped by research location (*n* = 27).

Supplementary Figure 10 Overview of the effects of sex and age influence on outcomes for patients treated with γδ T-cell immunotherapy (*n* = 138). **(A)** Proportions of treatment outcomes in male and female patients (male, *n* = 81; female, *n* = 57). Pie charts show the proportions of complete response (CR) and partial response (PR) within objective response (OR) patients of each sex (male, *n* = 14; female, *n* = 9). **(B)** Proportions of treatment outcomes in different age groups (≤ 60 years, *n* = 67; > 60 years, *n* = 71). Pie charts show the proportions of CR and PR within the OR patients of each age group (≤ 60 years, *n* = 16; > 60 years, *n* = 7). *p*-values (Pearson’s chi-squared test) are indicated in **(A, B)**. To take full advantage of these previous human studies, there were 138 patients for whom detailed information, such as age and sex, was available alongside treatment responses. The overall treatment responses were different among the three strategies (*p* = 0.000, Pearson’s chi-squared test, *n* = 138; **Table 3**), but, in this smaller sample, adoptive cell transfer (ACT) combined with other treatments except for IL-2 treatment (ACT+) had the best response rate [38% OR rate vs. 11% of *in vivo* stimulation patients (*p* = 0.013) and 2% of ACT patients (*p* = 0.000)]. Of these 138 patients, 57 were female and 81 were male. The responses [OR, SD, and progressive disease (PD)] to different treatments were similar when compared between sexes (*p* = 0.214, Pearson’s chi-squared test, *n* = 138; **Table 3**), whereas the age distribution was different between female and male patients (*p* = 0.008, Wilcoxon rank-sum test), with 61% and 40% of female and male patients less than 60 years old, respectively (median age: 57 years for females and 63 years for males). In addition, previous studies indicated that the percentage of Vγ9Vδ2 T cells was lower in elderly people and showed defects in functioning (92, 104); for this reason, the patients here were divided into age groups of ≤ 60 years and > 60 years (in order to have similar sample sizes in each group) to give a general idea of the effect of age on treatment response. There were no response, treatment type, or tumor type differences between these age groups (*n* = 138; **Table 3**). In addition, when comparing tumor types (hematological vs. solid), this smaller sample followed the same trend as the *n* = 307 sample presented above (**Figure 2B**; **Table 2**), in which both responses and treatment types were different between blood and solid tumors (**Table 3**; *n* = 138). This may emphasize the importance of different γδ T-cell therapy strategies on treatment outcomes.

## References

[1] Chien Y-h Meyer C Bonneville M. γδ T cells: first line of defense and beyond. Annu Rev Immunol (2014) 32:121–55. doi: 10.1146/annurev-immunol-032713-120216 24387714

[2] Papadopoulou M Sanchez Sanchez G Vermijlen D. Innate and adaptive γδ T cells: How, when, and why. Immunol Rev (2020) 298(1):99–116. doi: 10.1111/imr.12926 33146423

[3] Saura-Esteller J de Jong M King LA Ensing E Winograd B de Gruijl TD. Gamma delta T-cell based cancer immunotherapy: Past-Present-Future. Front Immunol (2022) 13:915837. doi: 10.3389/fimmu.2022.915837 35784326 PMC9245381

[4] Nielsen MM Witherden DA Havran WL. γδ T cells in homeostasis and host defence of epithelial barrier tissues. Nat Rev Immunol (2017) 17(12):733–45. doi: 10.1038/nri.2017.101 PMC5771804 28920588

[5] Silva-Santos B Serre K Norell H. γδ T cells in cancer. Nat Rev Immunol (2015) 15(11):683–91. doi: 10.1038/nri3904 26449179

[6] Laplagne C Ligat L Foote J Lopez F Fournié FJ Laurent C. Self-activation of Vγ9Vδ2 T cells by exogenous phosphoantigens involves TCR and butyrophilins. Cell Mol Immunol (2021) 18(8):1861–70. doi: 10.1038/s41423-021-00720-w PMC8237548 34183807

[7] Gober H-J Kistowska M Angman L Jenö P Mori L De Libero G. Human T cell receptor gammadelta cells recognize endogenous mevalonate metabolites in tumor cells. J Exp Med (2003) 197(2):163–8. doi: 10.1084/jem.20021500 PMC2193814 12538656

[8] Rigau M Ostrouska S Fulford TS Johnson DN Woods K Ruan Z. Butyrophilin 2A1 is essential for phosphoantigen reactivity by γδ T cells. Science. (2020) 367(6478):eaay5516. doi: 10.1126/science.aay5516 31919129

[9] Karunakaran MM Willcox CR Salim M Paletta D Fichtner AS Noll A. Butyrophilin-2A1 directly binds germline-encoded regions of the Vγ9Vδ2 TCR and is essential for phosphoantigen sensing. Immunity. (2020) 52(3):487–498.e6. doi: 10.1016/j.immuni.2020.02.014 32155411 PMC7083227

[10] Cano CE Pasero C de Gassart A Kerneur C Gabriac M Fullana M. BTN2A1, an immune checkpoint targeting Vγ9Vδ2 T cell cytotoxicity against malignant cells. Cell Rep (2021) 36(2):109359. doi: 10.1016/j.celrep.2021.109359 34260935

[11] Hayday AC. γδ T cell update: Adaptate orchestrators of immune surveillance. J Immunol (2019) 203(2):311–20. doi: 10.4049/jimmunol.1800934 31285310

[12] Vermijlen D Gatti D Kouzeli A Rus T Eberl M. γδ T cell responses: How many ligands will it take till we know? Semin Cell Dev Biol (2018) 84:75–86. doi: 10.1016/j.semcdb.2017.10.009 29402644

[13] Willcox BE Willcox CR. γδ TCR ligands: the quest to solve a 500-million-year-old mystery. Nat Immunol (2019) 20(2):121–8. doi: 10.1038/s41590-018-0304-y 30664765

[14] Willcox CR Vantourout P Salim M Zlatareva I Melandri D Zanardo L. Butyrophilin-like 3 directly binds a human Vγ4+ T cell receptor using a modality distinct from clonally-restricted antigen. Immunity. (2019) 51(5):813–825.e4. doi: 10.1016/j.immuni.2019.09.006 31628053 PMC6868513

[15] Herrmann T Karunakaran MM Fichtner AS. A glance over the fence: Using phylogeny and species comparison for a better understanding of antigen recognition by human γδ T-cells. Immunol Rev (2020) 298(1):218–36. doi: 10.1111/imr.12919 32981055

[16] Wegrecki M Ocampo TA Gunasinghe SD von Borstel A Tin SY Reijneveld JF. Atypical sideways recognition of CD1a by autoreactive γδ T cell receptors. Nat Commun (2022) 13(1):3872. doi: 10.1038/s41467-022-31443-9 35790773 PMC9256601

[17] Willcox CR Pitard V Netzer S Couzi L Salim M Silberzahn T. Cytomegalovirus and tumor stress surveillance by binding of a human γδ T cell antigen receptor to endothelial protein c receptor. Nat Immunol (2012) 13(9):872–9. doi: 10.1038/ni.2394 22885985

[18] Bruder J Siewert K Obermeier B Malotka J Scheinert P Kellermann J. Target specificity of an autoreactive pathogenic human γδ-T cell receptor in myositis. J Biol Chem (2012) 287(25):20986–95. doi: 10.1074/jbc.M112.356709 PMC3375522 22549773

[19] Vantourout P Hayday A. Six-of-the-best: unique contributions of γδ T cells to immunology. Nat Rev Immunol (2013) 13(2):88–100. doi: 10.1038/nri3384 23348415 PMC3951794

[20] Gavlovsky P-J Tonnerre P Guitton C Charreau B. Expression of MHC class I-related molecules MICA, HLA-e and EPCR shape endothelial cells with unique functions in innate and adaptive immunity. Hum Immunol (2016) 77(11):1084–91. doi: 10.1016/j.humimm.2016.02.007 26916837

[21] Pistoia V Tumino N Vacca P Veneziani I Moretta A Locatelli F. Human γδ T-cells: From surface receptors to the therapy of high-risk leukemias. Front Immunol (2018) 9:984. doi: 10.3389/fimmu.2018.00984 29867961 PMC5949323

[22] Pietschmann K Beetz S Welte S Martens I Gruen J Oberg HH. Toll-like receptor expression and function in subsets of human gammadelta T lymphocytes. Scand J Immunol (2009) 70(3):245–55. doi: 10.1111/j.1365-3083.2009.02290.x 19703014

[23] Angelini DF Borsellino G Poupot M Diamantini A Poupot R Bernardi G. FcgammaRIII discriminates between 2 subsets of Vgamma9Vdelta2 effector cells with different responses and activation pathways. Blood. (2004) 104(6):1801–7. doi: 10.1182/blood-2004-01-0331 15178578

[24] Hu W Shang R Yang J Chen C Liu Z Liang G. Skin γδ T cells and their function in wound healing. Front Immunol (2022) 13:875076. doi: 10.3389/fimmu.2022.875076 35479079 PMC9035842

[25] Xu W Lau ZWX Fulop T Larbi A. The aging of γδ T cells. Cells. (2020) 9(5):1181. doi: 10.3390/cells9051181 32397491 PMC7290956

[26] Ribot JC Lopes N Silva-Santos B. γδ T cells in tissue physiology and surveillance. Nat Rev Immunol (2021) 21(4):221–32. doi: 10.1038/s41577-020-00452-4 33057185

[27] Silva-Santos B Mensurado S Coffelt SB. γδ T cells: pleiotropic immune effectors with therapeutic potential in cancer. Nat Rev Cancer (2019) 19(7):392–404. doi: 10.1038/s41568-019-0153-5 31209264 PMC7614706

[28] Adams EJ Gu S Luoma AM. Human gamma delta T cells: Evolution and ligand recognition. Cell Immunol (2015) 296(1):31–40. doi: 10.1016/j.cellimm.2015.04.008 25991474 PMC4466157

[29] Fichtner AS Karunakaran MM Starick L Truman RW Herrmann T. The armadillo (Dasypus novemcinctus): A witness but not a functional example for the emergence of the butyrophilin 3/Vγ9Vδ2 system in placental mammals. Front Immunol (2018) 9:265. doi: 10.3389/fimmu.2018.00265 29527206 PMC5829056

[30] Karunakaran MM Göbel TW Starick L Walter L Herrmann T. Vγ9 and Vδ2 T cell antigen receptor genes and butyrophilin 3 (BTN3) emerged with placental mammals and are concomitantly preserved in selected species like alpaca (Vicugna pacos). Immunogenetics. (2014) 66(4):243–54. doi: 10.1007/s00251-014-0763-8 24526346

[31] Allaoui R Hagerling C Desmond E Warfvinge C-F Jirström K Leandersson K. Infiltration of γ⁢δ T cells, IL-17+ T cells and FoxP3+ T cells in human breast cancer. Cancer biomark (2017) 20(4):395–409. doi: 10.3233/CBM-170026 29060923 PMC5814667

[32] Girard P Charles J Cluzel C Degeorges E Manches O Plumas J. The features of circulating and tumor-infiltrating γδ T cells in melanoma patients display critical perturbations with prognostic impact on clinical outcome. Oncoimmunology. (2019) 8(8):1601483. doi: 10.1080/2162402X.2019.1601483 31413911 PMC6682366

[33] Pawlik-Gwozdecka D Zieliński M Sakowska J Adamkiewicz-Drożyńska E Trzonkowski P Niedźwiecki M. CD8+ γδ T cells correlate with favorable prognostic factors in childhood acute lymphoblastic leukemia. Arch Med Sci (2021) 17(2):561–3. doi: 10.5114/aoms/132316 PMC7959043 33747294

[34] Bruni E Cimino MM Donadon M Carriero R Terzoli S Piazza R. Intrahepatic CD69+Vδ1 T cells re-circulate in the blood of patients with metastatic colorectal cancer and limit tumor progression. J Immunother Cancer (2022) 10(7):e004579. doi: 10.1136/jitc-2022-004579 35863820 PMC9310256

[35] Wu Y Biswas D Usaite I Angelova M Boeing S Karasaki T. A local human Vδ1 T cell population is associated with survival in nonsmall-cell lung cancer. Nat Cancer (2022) 3(6):696–709. doi: 10.1038/s43018-022-00376-z 35637401 PMC9236901

[36] Ma C Zhang Q Ye J Wang F Zhang Y Wevers E. Tumor-infiltrating γδ T lymphocytes predict clinical outcome in human breast cancer. J Immunol (2012) 189(10):5029–36. doi: 10.4049/jimmunol.1201892 PMC4832413 23034170

[37] Nguyen S Chevalier MF Benmerzoug S Cesson V Schneider AK Rodrigues-Dias S. Vδ2 T cells are associated with favorable clinical outcomes in patients with bladder cancer and their tumor reactivity can be boosted by BCG and zoledronate treatments. J Immunother Cancer (2022) 10(8):e004880. doi: 10.1136/jitc-2022-004880 36002184 PMC9413168

[38] Godder KT Henslee-Downey PJ Mehta J Park BS Chiang KY Abhyankar S. Long term disease-free survival in acute leukemia patients recovering with increased gammadelta T cells after partially mismatched related donor bone marrow transplantation. Bone Marrow Transplant (2007) 39(12):751–7. doi: 10.1038/sj.bmt.1705650 17450185

[39] Gentles AJ Newman AM Liu CL Bratman SV Feng W Kim D. The prognostic landscape of genes and infiltrating immune cells across human cancers. Nat Med (2015) 21(8):938–45. doi: 10.1038/nm.3909 PMC4852857 26193342

[40] Lamb LS Henslee-Downey PJ Parrish RS Godder K Thompson J Lee C. Increased frequency of TCR gamma delta + T cells in disease-free survivors following T cell-depleted, partially mismatched, related donor bone marrow transplantation for leukemia. J Hematother (1996) 5(5):503–9. doi: 10.1089/scd.1.1996.5.503 8938522

[41] Perko R Kang G Sunkara A Leung W Thomas PG Dallas MH. Gamma delta T cell reconstitution is associated with fewer infections and improved event-free survival after hematopoietic stem cell transplantation for pediatric leukemia. Biol Blood Marrow Transplant (2015) 21(1):130–6. doi: 10.1016/j.bbmt.2014.09.027 PMC4288038 25445640

[42] Jin Z Luo Q Lu S Wang X He Z Lai J. Oligoclonal expansion of TCR vδ T cells may be a potential immune biomarker for clinical outcome of acute myeloid leukemia. J Hematol Oncol (2016) 9(1):126. doi: 10.1186/s13045-016-0353-3 27863523 PMC5116135

[43] Kong X Zheng J Liu X Wang W Jiang X Chen J. High TRGV 9 subfamily expression marks an improved overall survival in patients with acute myeloid leukemia. Front Immunol (2022) 13:823352. doi: 10.3389/fimmu.2022.823352 35222403 PMC8866455

[44] Coscia M Vitale C Peola S Foglietta M Rigoni M Griggio V. Dysfunctional Vγ9Vδ2 T cells are negative prognosticators and markers of dysregulated mevalonate pathway activity in chronic lymphocytic leukemia cells. Blood. (2012) 120(16):3271–9. doi: 10.1182/blood-2012-03-417519 22932792

[45] Molina-Aguilar R Montiel-Cervantes LA Anguiano-Peñaloza SV Lezama R Vela-Ojeda J Reyes-Maldonado E. γδ T cells number, CD200, and Flt3 expression is associated with higher progression free survival in patients with chronic myeloid leukemia. Arch Med Res (2020) 51(3):194–203. doi: 10.1016/j.arcmed.2020.01.013 32113783

[46] Kobayashi H Tanaka Y Nakazawa H Yagi J Minato N Tanabe K. A new indicator of favorable prognosis in locally advanced renal cell carcinomas: gamma delta T-cells in peripheral blood. Anticancer Res (2011) 31(3):1027–31. 21498733

[47] Wistuba-Hamprecht K Martens A Haehnel K Foppen MG Yuan J Postow MA. Proportions of blood-borne Vδ1+ and Vδ2+ T-cells are associated with overall survival of melanoma patients treated with ipilimumab. Eur J Cancer (2016) 64:116–26. doi: 10.1016/j.ejca.2016.06.001 PMC5201188 27400322

[48] Gherardin NA Waldeck K Caneborg A Martelotto LG Balachander S Zethoven M. γδ T cells in merkel cell carcinomas have a proinflammatory profile prognostic of patient survival. Cancer Immunol Res (2021) 9(6):612–23. doi: 10.1158/2326-6066.CIR-20-0817 33674358

[49] Raspollini MR Castiglione F Rossi Degl'innocenti D Amunni G Villanucci A Garbini F. Tumour-infiltrating gamma/delta T-lymphocytes are correlated with a brief disease-free interval in advanced ovarian serous carcinoma. Ann Oncol (2005) 16(4):590–6. doi: 10.1093/annonc/mdi112 15699022

[50] Lu H Dai W Guo J Wang D Wen S Yang L. High abundance of intratumoral γδ T cells favors a better prognosis in head and neck squamous cell carcinoma: A bioinformatic analysis. Front Immunol (2020) 11:573920. doi: 10.3389/fimmu.2020.573920 33101298 PMC7555127

[51] Wu Y Kyle-Cezar F Woolf RT Naceur-Lombardelli C Owen J Biswas D. An innate-like Vδ1+ γδ T cell compartment in the human breast is associated with remission in triple-negative breast cancer. Sci Transl Med (2019) 11(513):eaax9364. doi: 10.1126/scitranslmed.aax9364 31597756 PMC6877350

[52] Boissière-Michot F Chabab G Mollevi C Guiu S Lopez-Crapez E Ramos J. Clinicopathological correlates of γδ T cell infiltration in triple-negative breast cancer. Cancers (Basel) (2021) 13(4):765. doi: 10.3390/cancers13040765 33673133 PMC7918092

[53] Bense RD Sotiriou C Piccart-Gebhart MJ Haanen JBAG van Vugt MATM de Vries EGE. Relevance of tumor-infiltrating immune cell composition and functionality for disease outcome in breast cancer. J Natl Cancer Inst (2017) 109(1):djw192. doi: 10.1093/jnci/djw192 27737921 PMC6284248

[54] Zheng S Zou Y Xie X Liang J Yang A Yu K. Development and validation of a stromal immune phenotype classifier for predicting immune activity and prognosis in triple-negative breast cancer. Int J Cancer (2020) 147(2):542–53. doi: 10.1002/ijc.33009 32285442

[55] Chobrutskiy A Chobrutskiy BI Zaman S Hsiang M Blanck G. Chemical features of blood-borne TRG CDR3s associated with an increased overall survival in breast cancer. Breast Cancer Res Treat (2021) 185(3):591–600. doi: 10.1007/s10549-020-05996-6 33180235

[56] Katsuta E Qi Q Peng X Hochwald SN Yan L Takabe K. Pancreatic adenocarcinomas with mature blood vessels have better overall survival. Sci Rep (2019) 9(1):1310. doi: 10.1038/s41598-018-37909-5 30718678 PMC6362082

[57] Chen Q Pu N Yin H Zhang J Zhao G Lou W. CD73 acts as a prognostic biomarker and promotes progression and immune escape in pancreatic cancer. J Cell Mol Med (2020) 24(15):8674–86. doi: 10.1111/jcmm.15500 PMC7412695 32643277

[58] Chitadze G Oberg H-H Wesch D Kabelitz D. The ambiguous role of γδ T lymphocytes in antitumor immunity. Trends Immunol (2017) 38(9):668–78. doi: 10.1016/j.it.2017.06.004 28709825

[59] Raverdeau M Cunningham SP Harmon C Lynch L. γδ T cells in cancer: a small population of lymphocytes with big implications. Clin Transl Immunol (2019) 8(10):e01080. doi: 10.1002/cti2.1080 PMC6787154 31624593

[60] Li Y Li G Zhang J Wu X Chen X. The dual roles of human γδ T cells: Anti-tumor or tumor-promoting. Front Immunol (2020) 11:619954. doi: 10.3389/fimmu.2020.619954 33664732 PMC7921733

[61] Chabab G Barjon C Bonnefoy N Lafont V. Pro-tumor γδ T cells in human cancer: Polarization, mechanisms of action, and implications for therapy. Front Immunol (2020) 11:2186. doi: 10.3389/fimmu.2020.02186 33042132 PMC7524881

[62] Wu P Wu D Ni C Ye J Chen W Hu G. γδT17 cells promote the accumulation and expansion of myeloid-derived suppressor cells in human colorectal cancer. Immunity. (2014) 40(5):785–800. doi: 10.1016/j.immuni.2014.03.013 24816404 PMC4716654

[63] Jin C Lagoudas GK Zhao C Bullman S Bhutkar A Hu B. Commensal microbiota promote lung cancer development *via* γδ T cells. Cell. (2019) 176(5):998–1013.e16. doi: 10.1016/j.cell.2018.12.040 30712876 PMC6691977

[64] Daley D Zambirinis CP Seifert L Akkad N Mohan N Werba G. γδ T cells support pancreatic oncogenesis by restraining αβ T cell activation. Cell. (2016) 166(6):1485–1499.e15. doi: 10.1016/j.cell.2016.07.046 27569912 PMC5017923

[65] Reis BS Darcy PW Khan IZ Moon CS Kornberg AE Schneider VS. TCR-vγδ usage distinguishes protumor from antitumor intestinal γδ T cell subsets. Science. (2022) 377(6603):276–84. doi: 10.1126/science.abj8695 PMC9326786 35857588

[66] Singh AK McGuirk JP. CAR T cells: continuation in a revolution of immunotherapy. Lancet Oncol (2020) 21(3):e168–78. doi: 10.1016/S1470-2045(19)30823-X 32135120

[67] Mingari MC Varese P Bottino C Melioli G Moretta A Moretta L. Clonal analysis of CD4-CD8- human thymocytes expressing a T cell receptor gamma/delta chain. direct evidence for the *de novo* expression of CD8 surface antigen and of cytolytic activity against tumor targets. Eur J Immunol (1988) 18(11):1831–4. doi: 10.1002/eji.1830181127 2974427

[68] Wilhelm M Kunzmann V Eckstein S Reimer P Weissinger F Ruediger T. Gammadelta T cells for immune therapy of patients with lymphoid malignancies. Blood. (2003) 102(1):200–6. doi: 10.1182/blood-2002-12-3665 12623838

[69] Morita CT Jin C Sarikonda G Wang H. Nonpeptide antigens, presentation mechanisms, and immunological memory of human Vgamma2Vdelta2 T cells: discriminating friend from foe through the recognition of prenyl pyrophosphate antigens. Immunol Rev (2007) 215:59–76. doi: 10.1111/j.1600-065X.2006.00479.x 17291279

[70] Kunzmann V Bauer E Feurle J Weissinger F Tony HP Wilhelm M. Stimulation of gammadelta T cells by aminobisphosphonates and induction of antiplasma cell activity in multiple myeloma. Blood (2000) 96(2):384–92. doi: 10.1182/blood.V96.2.384 10887096

[71] Almeida AR Correia DV Fernandes-Platzgummer A da Silva CL da Silva MG Anjos DR. Delta one T cells for immunotherapy of chronic lymphocytic leukemia: Clinical-grade Expansion/Differentiation and preclinical proof of concept. Clin Cancer Res (2016) 22(23):5795–804. doi: 10.1158/1078-0432.CCR-16-0597 27307596

[72] Papapetrou PD. Bisphosphonate-associated adverse events. Hormones (Athens) (2009) 8(2):96–110. doi: 10.14310/horm.2002.1226 19570737

[73] Dieli F Vermijlen D Fulfaro F Caccamo N Meraviglia S Cicero G. Targeting human {gamma}delta} T cells with zoledronate and interleukin-2 for immunotherapy of hormone-refractory prostate cancer. Cancer Res (2007) 67(15):7450–7. doi: 10.1158/0008-5472.CAN-07-0199 PMC3915341 17671215

[74] Lang JM Kaikobad MR Wallace M Staab MJ Horvath DL Wilding G. Pilot trial of interleukin-2 and zoledronic acid to augment γδ T cells as treatment for patients with refractory renal cell carcinoma. Cancer Immunol Immunother (2011) 60(10):1447–60. doi: 10.1007/s00262-011-1049-8 PMC3177972 21647691

[75] Kobayashi H Tanaka Y Yagi J Osaka Y Nakazawa H Uchiyama T. Safety profile and anti-tumor effects of adoptive immunotherapy using gamma-delta T cells against advanced renal cell carcinoma: a pilot study. Cancer Immunol Immunother (2007) 56(4):469–76. doi: 10.1007/s00262-006-0199-6 PMC11030814 16850345

[76] Bennouna J Bompas E Neidhardt EM Rolland F Philip I Galéa C. Phase-I study of innacell gammadelta, an autologous cell-therapy product highly enriched in gamma9delta2 T lymphocytes, in combination with IL-2, in patients with metastatic renal cell carcinoma. Cancer Immunol Immunother (2008) 57(11):1599–609. doi: 10.1007/s00262-008-0491-8 PMC11030608 18301889

[77] Abe Y Muto M Nieda M Nakagawa Y Nicol A Kaneko T. Clinical and immunological evaluation of zoledronate-activated Vgamma9gammadelta T-cell-based immunotherapy for patients with multiple myeloma. Exp Hematol (2009) 37(8):956–68. doi: 10.1016/j.exphem.2009.04.008 19409955

[78] Andreu-Ballester JC Galindo-Regal L Hidalgo-Coloma J Cuéllar C García-Ballesteros C Hurtado C. Differences in circulating γδ T cells in patients with primary colon cancer and relation with prognostic factors. PloS One (2020) 15(12):e0243545. doi: 10.1371/journal.pone.0243545 33326443 PMC7743935

[79] Burnham RE Zoine JT Story JY Garimalla SN Gibson G Rae A. Characterization of donor variability for γδ T cell ex vivo expansion and development of an allogeneic γδ T cell immunotherapy. Front Med (Lausanne) (2020) 7:588453. doi: 10.3389/fmed.2020.588453 33282892 PMC7691424

[80] Fichtner AS Ravens S Prinz I. Human γδ TCR repertoires in health and disease. Cells. (2020) 9(4):800. doi: 10.3390/cells9040800 32225004 PMC7226320

[81] Lamb LS Gee AP Hazlett LJ Musk P Parrish RS O'Hanlon TP. Influence of T cell depletion method on circulating gammadelta T cell reconstitution and potential role in the graft-versus-leukemia effect. Cytotherapy. (1999) 1(1):7–19. doi: 10.1080/0032472031000141295 19746645

[82] Xu Y Xiang Z Alnaggar M Kouakanou L Li J He J. Allogeneic Vγ9Vδ2 T-cell immunotherapy exhibits promising clinical safety and prolongs the survival of patients with late-stage lung or liver cancer. Cell Mol Immunol (2021) 18(2):427–39. doi: 10.1038/s41423-020-0515-7 PMC8027668 32939032

[83] Gassart A Le K-S Brune P Agaugué S Sims J Goubard A. Development of ICT01, a first-in-class, anti-BTN3A antibody for activating Vγ9Vδ2 T cell-mediated antitumor immune response. Sci Transl Med (2021) 13(616):eabj0835. doi: 10.1126/scitranslmed.abj0835 34669444

[84] He P Liu H Zimdahl B Wang J Luo M Chang Q. A novel antibody-TCR (AbTCR) T-cell therapy is safe and effective against CD19-positive relapsed/refractory b-cell lymphoma. J Cancer Res Clin Oncol (2022). doi: 10.1007/s00432-022-04132-9 PMC11797221 35776199

[85] Bennouna J Levy V Sicard H Senellart H Audrain M Hiret S. Phase I study of bromohydrin pyrophosphate (BrHPP, IPH 1101), a Vgamma9Vdelta2 T lymphocyte agonist in patients with solid tumors. Cancer Immunol Immunother (2010) 59(10):1521–30. doi: 10.1007/s00262-010-0879-0 PMC11030967 20563721

[86] Meraviglia S Eberl M Vermijlen D Todaro M Buccheri S Cicero G. *In vivo* manipulation of Vgamma9Vdelta2 T cells with zoledronate and low-dose interleukin-2 for immunotherapy of advanced breast cancer patients. Clin Exp Immunol (2010) 161(2):290–7. doi: 10.1111/j.1365-2249.2010.04167.x PMC2909411 20491785

[87] Nakajima J Murakawa T Fukami T Goto S Kaneko T Yoshida Y. A phase I study of adoptive immunotherapy for recurrent non-small-cell lung cancer patients with autologous gammadelta T cells. Eur J Cardiothorac Surg (2010) 37(5):1191–7. doi: 10.1016/j.ejcts.2009.11.051 20137969

[88] Kobayashi H Tanaka Y Yagi J Minato N Tanabe K. Phase I/II study of adoptive transfer of γδ T cells in combination with zoledronic acid and IL-2 to patients with advanced renal cell carcinoma. Cancer Immunol Immunother (2011) 60(8):1075–84. doi: 10.1007/s00262-011-1021-7 PMC11029699 21519826

[89] Nicol AJ Tokuyama H Mattarollo SR Hagi T Suzuki K Yokokawa K. Clinical evaluation of autologous gamma delta T cell-based immunotherapy for metastatic solid tumours. Br J Cancer (2011) 105(6):778–86. doi: 10.1038/bjc.2011.293 PMC3171009 21847128

[90] Sakamoto M Nakajima J Murakawa T Fukami T Yoshida Y Murayama T. Adoptive immunotherapy for advanced non-small cell lung cancer using zoledronate-expanded γδTcells: a phase I clinical study. J Immunother (2011) 34(2):202–11. doi: 10.1097/CJI.0b013e318207ecfb 21304399

[91] Noguchi A Kaneko T Kamigaki T Fujimoto K Ozawa M Saito M. Zoledronate-activated Vγ9γδ T cell-based immunotherapy is feasible and restores the impairment of γδ T cells in patients with solid tumors. Cytotherapy. (2011) 13(1):92–7. doi: 10.3109/14653249.2010.515581 20831354

[92] Kunzmann V Smetak M Kimmel B Weigang-Koehler K Goebeler M Birkmann J. Tumor-promoting versus tumor-antagonizing roles of γδ T cells in cancer immunotherapy: results from a prospective phase I/II trial. J Immunother (2012) 35(2):205–13. doi: 10.1097/CJI.0b013e318245bb1e 22306909

[93] Izumi T Kondo M Takahashi T Fujieda N Kondo A Tamura N. Ex vivo characterization of γδ T-cell repertoire in patients after adoptive transfer of Vγ9Vδ2 T cells expressing the interleukin-2 receptor β-chain and the common γ-chain. Cytotherapy. (2013) 15(4):481–91. doi: 10.1016/j.jcyt.2012.12.004 23391461

[94] Wilhelm M Smetak M Schaefer-Eckart K Kimmel B Birkmann J Einsele H. Successful adoptive transfer and *in vivo* expansion of haploidentical γδ T cells. J Transl Med (2014) 12:45. doi: 10.1186/1479-5876-12-45 24528541 PMC3926263

[95] Wada I Matsushita H Noji S Mori K Yamashita H Nomura S. Intraperitoneal injection of *in vitro* expanded Vγ9Vδ2 T cells together with zoledronate for the treatment of malignant ascites due to gastric cancer. Cancer Med (2014) 3(2):362–75. doi: 10.1002/cam4.196 PMC3987085 24515916

[96] Cui J Li L Wang C Jin H Yao C Wang Y. Combined cellular immunotherapy and chemotherapy improves clinical outcome in patients with gastric carcinoma. Cytotherapy. (2015) 17(7):979–88. doi: 10.1016/j.jcyt.2015.03.605 25890480

[97] Pressey JG Adams J Harkins L Kelly D You Z Lamb LS. *In vivo* expansion and activation of γδ T cells as immunotherapy for refractory neuroblastoma: A phase 1 study. Med (Baltimore) (2016) 95(39):e4909. doi: 10.1097/MD.0000000000004909 PMC5265919 27684826

[98] Aoki T Matsushita H Hoshikawa M Hasegawa K Kokudo N Kakimi K. Adjuvant combination therapy with gemcitabine and autologous γδ T-cell transfer in patients with curatively resected pancreatic cancer. Cytotherapy. (2017) 19(4):473–85. doi: 10.1016/j.jcyt.2017.01.002 28188072

[99] Sugie T Suzuki E Yamauchi A Yamagami K Masuda N Gondo N. Combined effects of neoadjuvant letrozole and zoledronic acid on γδT cells in postmenopausal women with early-stage breast cancer. Breast. (2018) 38:114–9. doi: 10.1016/j.breast.2017.12.017 29310035

[100] Lin M Zhang X Liang S Luo H Alnaggar M Liu A. Irreversible electroporation plus allogenic Vγ9Vδ2 T cells enhances antitumor effect for locally advanced pancreatic cancer patients. Signal Transduct Target Ther (2020) 5(1):215. doi: 10.1038/s41392-020-00260-1 33093457 PMC7582168

[101] Kakimi K Matsushita H Masuzawa K Karasaki T Kobayashi Y Nagaoka K. Adoptive transfer of zoledronate-expanded autologous Vγ9Vδ2 T-cells in patients with treatment-refractory non-small-cell lung cancer: a multicenter, open-label, single-arm, phase 2 study. J Immunother Cancer (2020) 8(2):e001185. doi: 10.1136/jitc-2020-001185 32948652 PMC7511646

[102] Fazzi R Petrini I Giuliani N Morganti R Carulli G Palma BD. Phase II trial of maintenance treatment with IL2 and zoledronate in multiple myeloma after bone marrow transplantation: Biological and clinical results. Front Immunol (2020) 11:573156. doi: 10.3389/fimmu.2020.573156 33613510 PMC7890401

[103] Zhang T Chen J Niu L Liu Y Ye G Jiang M. Clinical safety and efficacy of locoregional therapy combined with adoptive transfer of allogeneic γδ T cells for advanced hepatocellular carcinoma and intrahepatic cholangiocarcinoma. J Vasc Interv Radiol (2022) 33(1):19–27.e3. doi: 10.1016/j.jvir.2021.09.012 34600129

[104] Zheng Y Wang P-P Fu Y Chen Y-Y Ding Z-Y. Zoledronic acid enhances the efficacy of immunotherapy in non-small cell lung cancer. Int Immunopharmacol (2022) 110:109030. doi: 10.1016/j.intimp.2022.109030 35978519

[105] Lamb LS Bowersock J Dasgupta A Gillespie GY Su Y Johnson A. Engineered drug resistant γδ T cells kill glioblastoma cell lines during a chemotherapy challenge: a strategy for combining chemo- and immunotherapy. PloS One (2013) 8(1):e51805. doi: 10.1371/journal.pone.0051805 23326319 PMC3543433

[106] Strijker JGM Pscheid R Drent E van der Hoek JJF Koopmans B Ober K. αβ-T cells engineered to express γδ-T cell receptors can kill neuroblastoma organoids independent of MHC-I expression. J Pers Med (2021) 11(9):923. doi: 10.3390/jpm11090923 34575700 PMC8471928

[107] Straetemans T Kierkels GJJ Doorn R Jansen K Heijhuurs S Santos JMD. GMP-grade manufacturing of T cells engineered to express a defined γδTCR. Front Immunol (2018) 9:1062. doi: 10.3389/fimmu.2018.01062 29899740 PMC5988845

[108] Michishita Y Hirokawa M Guo Y-M Abe Y Liu J Ubukawa K. Age-associated alteration of γδ T-cell repertoire and different profiles of activation-induced death of Vδ1 and Vδ2 T cells. Int J Hematol (2011) 94(3):230–40. doi: 10.1007/s12185-011-0907-7 21858446

[109] Kallemeijn MJ Boots AMH van der Klift MY Brouwer E Abdulahad WH Verhaar JAN. Ageing and latent CMV infection impact on maturation, differentiation and exhaustion profiles of T-cell receptor gammadelta T-cells. Sci Rep (2017) 7(1):5509. doi: 10.1038/s41598-017-05849-1 28710491 PMC5511140

[110] Liu M Wang X Li W Yu X Flores-Villanueva P Xu-Monette ZY. Targeting PD-L1 in non-small cell lung cancer using CAR T cells. Oncogenesis. (2020) 9(8):72. doi: 10.1038/s41389-020-00257-z 32792499 PMC7426958

[111] Themeli M Kloss CC Ciriello G Fedorov VD Perna F Gonen M. Generation of tumor-targeted human T lymphocytes from induced pluripotent stem cells for cancer therapy. Nat Biotechnol (2013) 31(10):928–33. doi: 10.1038/nbt.2678 PMC5722218 23934177

[112] Zeng J Tang SY Wang S. Derivation of mimetic γδ T cells endowed with cancer recognition receptors from reprogrammed γδ T cell. PloS One (2019) 14(5):e0216815. doi: 10.1371/journal.pone.0216815 31071196 PMC6508724

[113] Deisher T Taylor M Ashok A Jarzyna P Zahid Y Lee-Diaz S. AVM0703, a new treatment option for lymphoma patients. Blood. (2019) 134(Supplement_1):5308. doi: 10.1182/blood-2019-128812

[114] Deisher T Sawas S Suwito K Rylatt C Jarzyna PA Zahid Y. AVM0703 tumor debulking enhances Cy/Flu efficacy. Blood. (2021) 138(Supplement 1):4498. doi: 10.1182/blood-2021-153990

[115] Formenti SC Rudqvist N-P Golden E Cooper B Wennerberg E Lhuillier C. Radiotherapy induces responses of lung cancer to CTLA-4 blockade. Nat Med (2018) 24(12):1845–51. doi: 10.1038/s41591-018-0232-2 PMC6286242 30397353

[116] Srivastava S Furlan SN Jaeger-Ruckstuhl CA Sarvothama M Berger C Smythe KS. Immunogenic chemotherapy enhances recruitment of CAR-T cells to lung tumors and improves antitumor efficacy when combined with checkpoint blockade. Cancer Cell (2021) 39(2):193–208.e10. doi: 10.1016/j.ccell.2020.11.005 33357452 PMC7878409

