# Supplementary figures and images for: A close look at current γδ T-cell immunotherapy
Source: Front Immunol. 2023 Mar 31;14:1140623. doi: 10.3389/fimmu.2023.1140623 (PMC10102511; doi:10.3389/fimmu.2023.1140623)

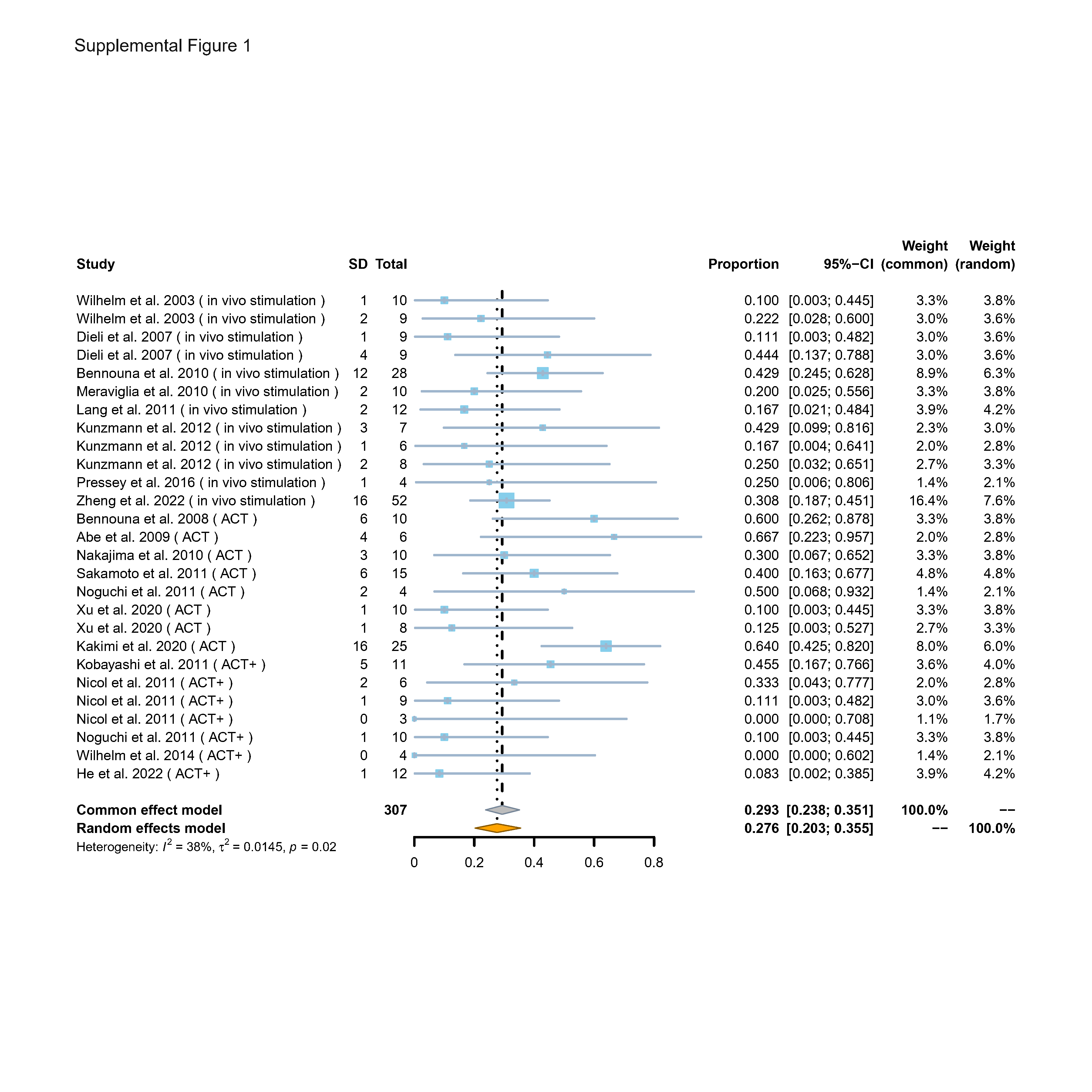

Supplement: Supplementary Figure 1 — Forest plot of stable disease (SD) rate (n = 27). SD indicates the number of patients in each cohort who achieved stable disease. The total indicates the total number of measurable patients in each cohort. The SD rate, 95% confidence interval (CI), and weights of fixed- and random-effects models are indicated for each cohort. Blue squares show the mean SD rate of each cohort, and the size indicates the weight of the cohort; gray lines show the 95% CI, and the diamond shapes show the pooled weighted means of the SD rate using fixed- and random-effects models. [file Image_1.tif]

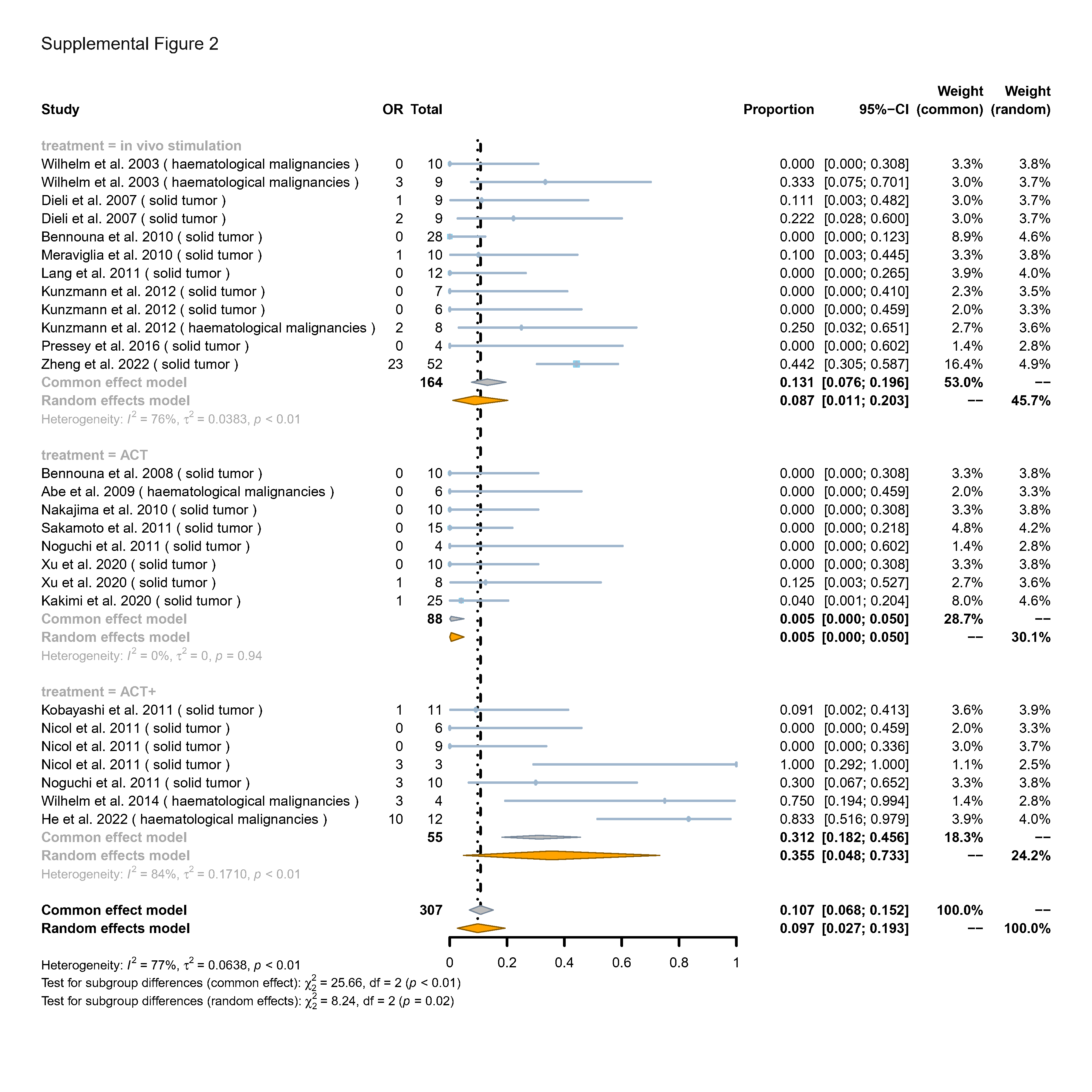

Supplement: Supplementary Figure 2 — Forest plot of objective response (OR) rate sub-grouped by treatment strategy (n = 27). The OR indicates the number of patients in each cohort who achieved an objective response. The total indicates the total number of measurable patients in each cohort. The OR rate, 95% confidence interval (CI), and weights of fixed- and random-effects models are indicated for each cohort. Blue squares show the mean OR rate of each cohort, and the size indicates the weight of the cohort; gray lines show the 95% CI, and the diamond shapes indicate the pooled weighted means of OR rates for each treatment subgroup and for all cohorts using fixed- and random-effects models. [file Image_2.tif]

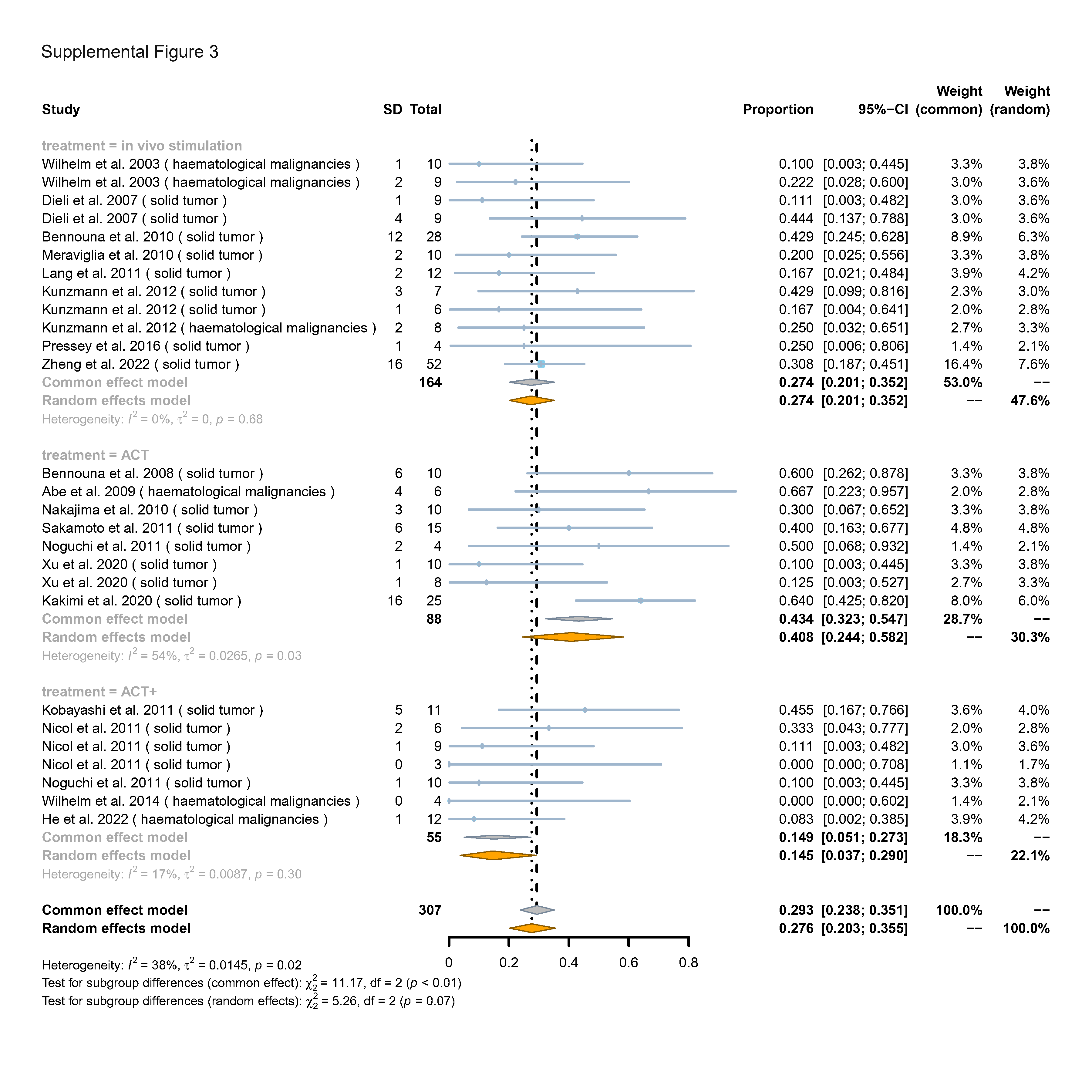

Supplement: Supplementary Figure 3 — Forest plot of stable disease (SD) rate subgrouped by treatment strategy (n = 27). [file Image_3.tif]

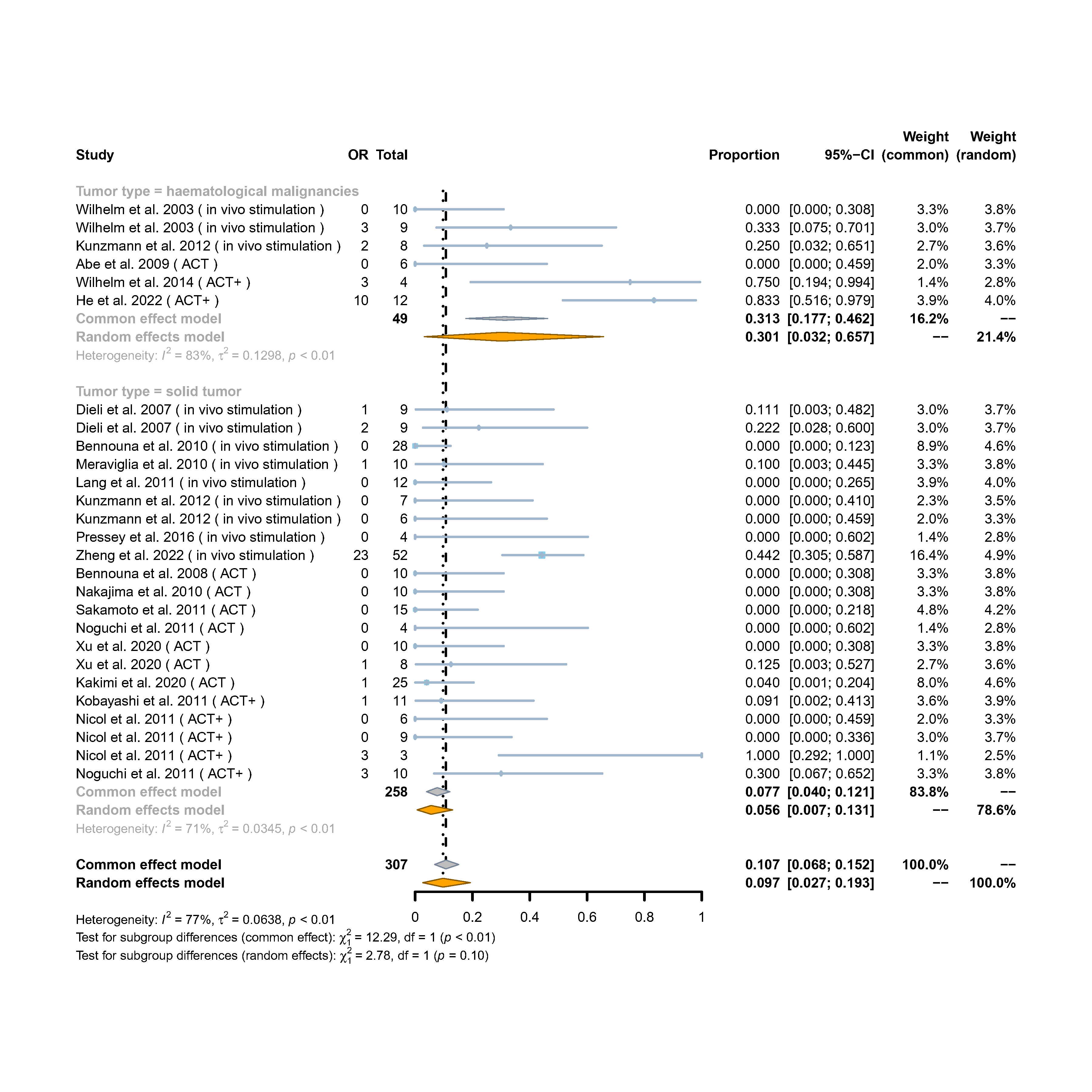

Supplement: Supplementary Figure 4 — Forest plot of objective response (OR) rate subgrouped by tumor type (n = 27). [file Image_4.tif]

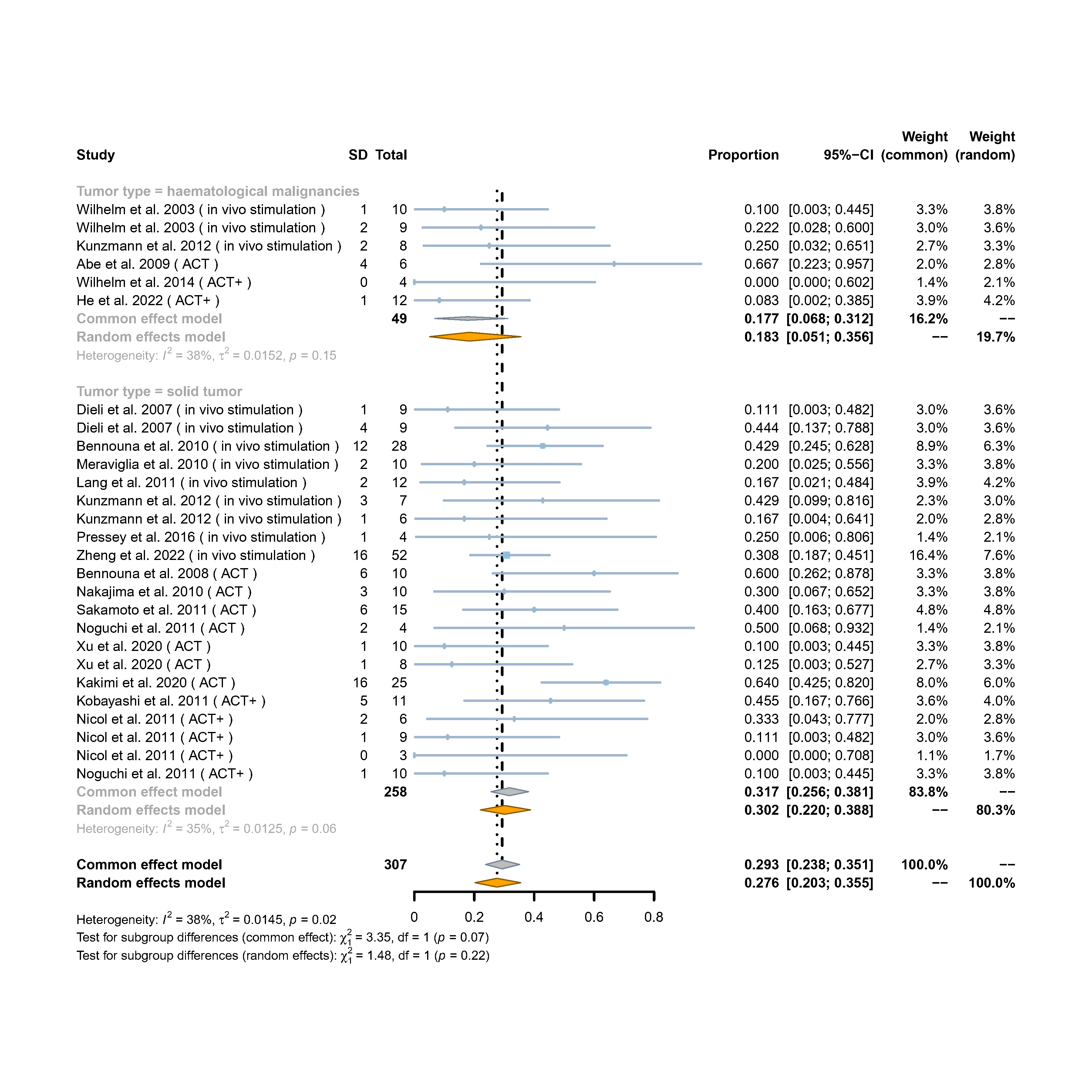

Supplement: Supplementary Figure 5 — Forest plot of stable disease (SD) rate subgrouped by tumor type (n = 27). [file Image_5.tif]

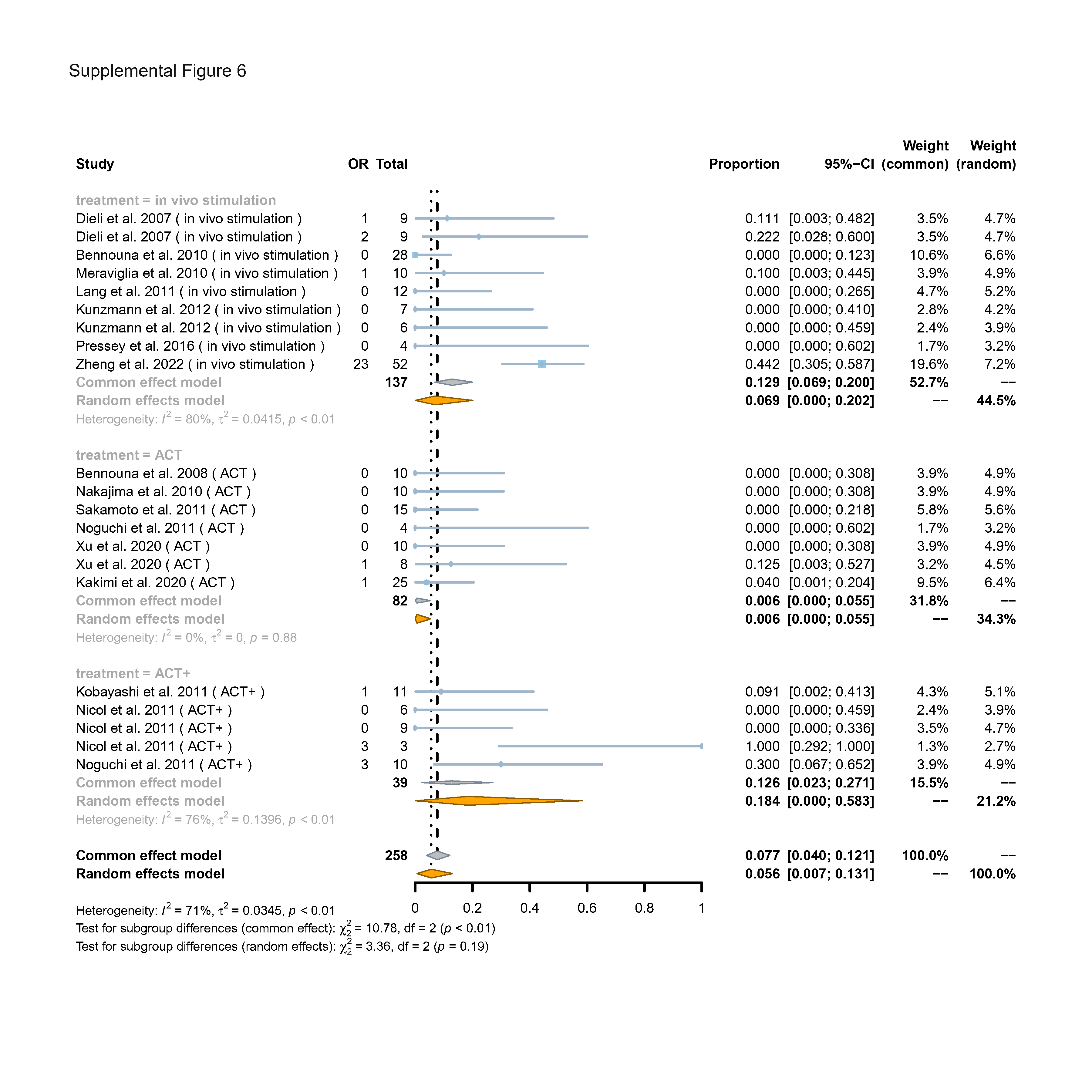

Supplement: Supplementary Figure 6 — Forest plot of objective response (OR) rate in solid tumor cohorts subgrouped by treatment (n = 21). [file Image_6.tif]

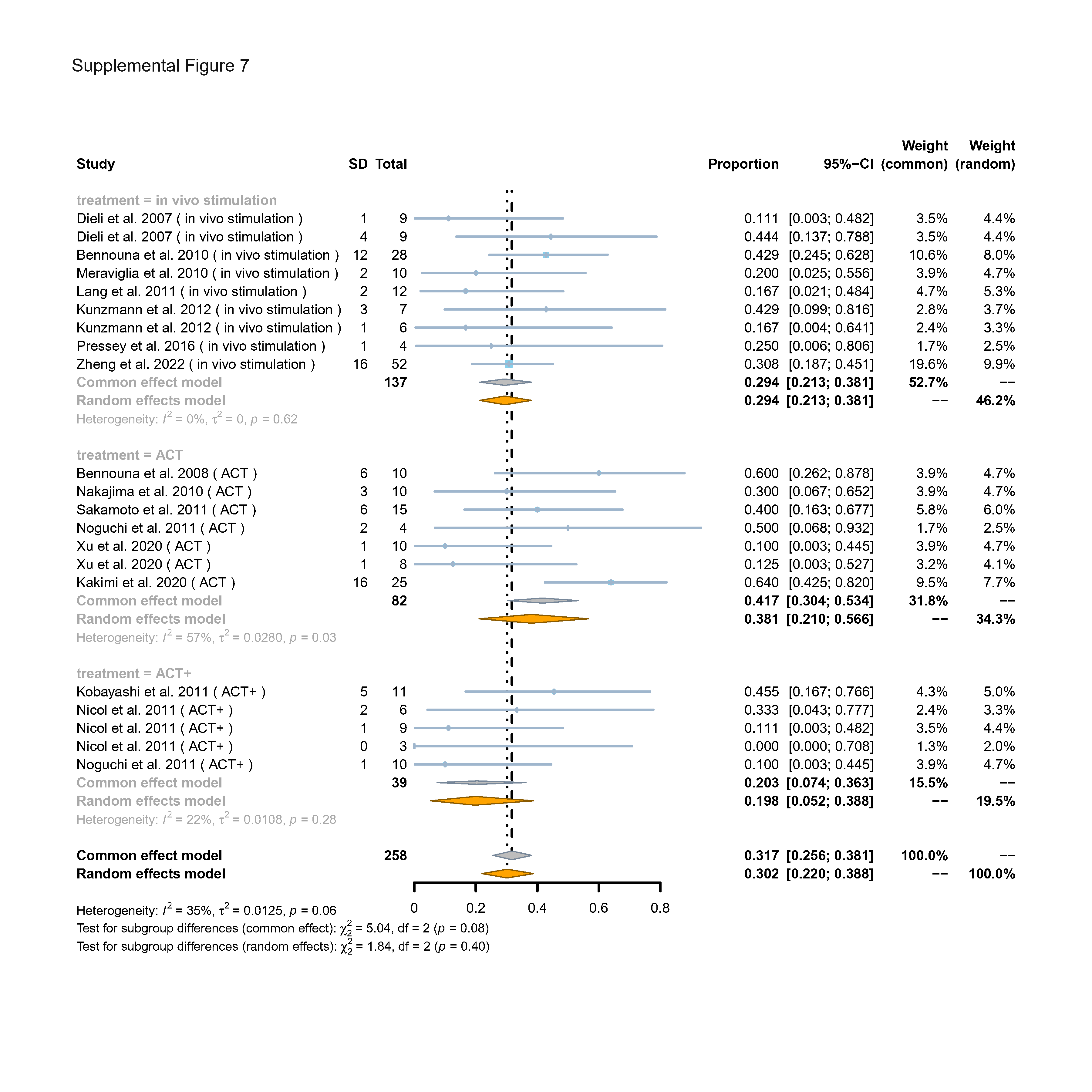

Supplement: Supplementary Figure 7 — Forest plot of stable disease (SD) rate in solid tumor cohorts subgrouped by treatment (n = 21). [file Image_7.tif]

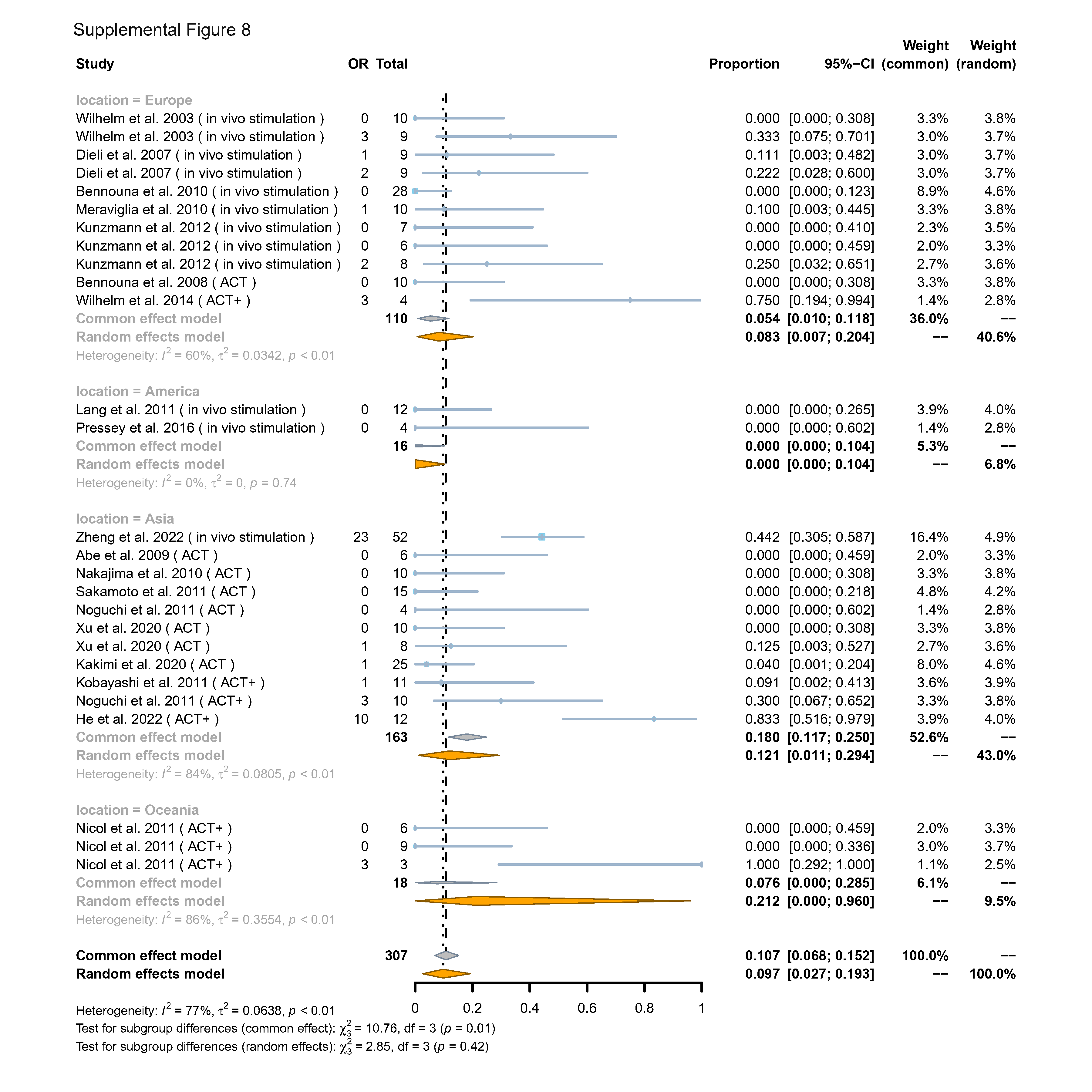

Supplement: Supplementary Figure 8 — Forest plot of objective response (OR) rate subgrouped by research location (n = 27). [file Image_8.tif]

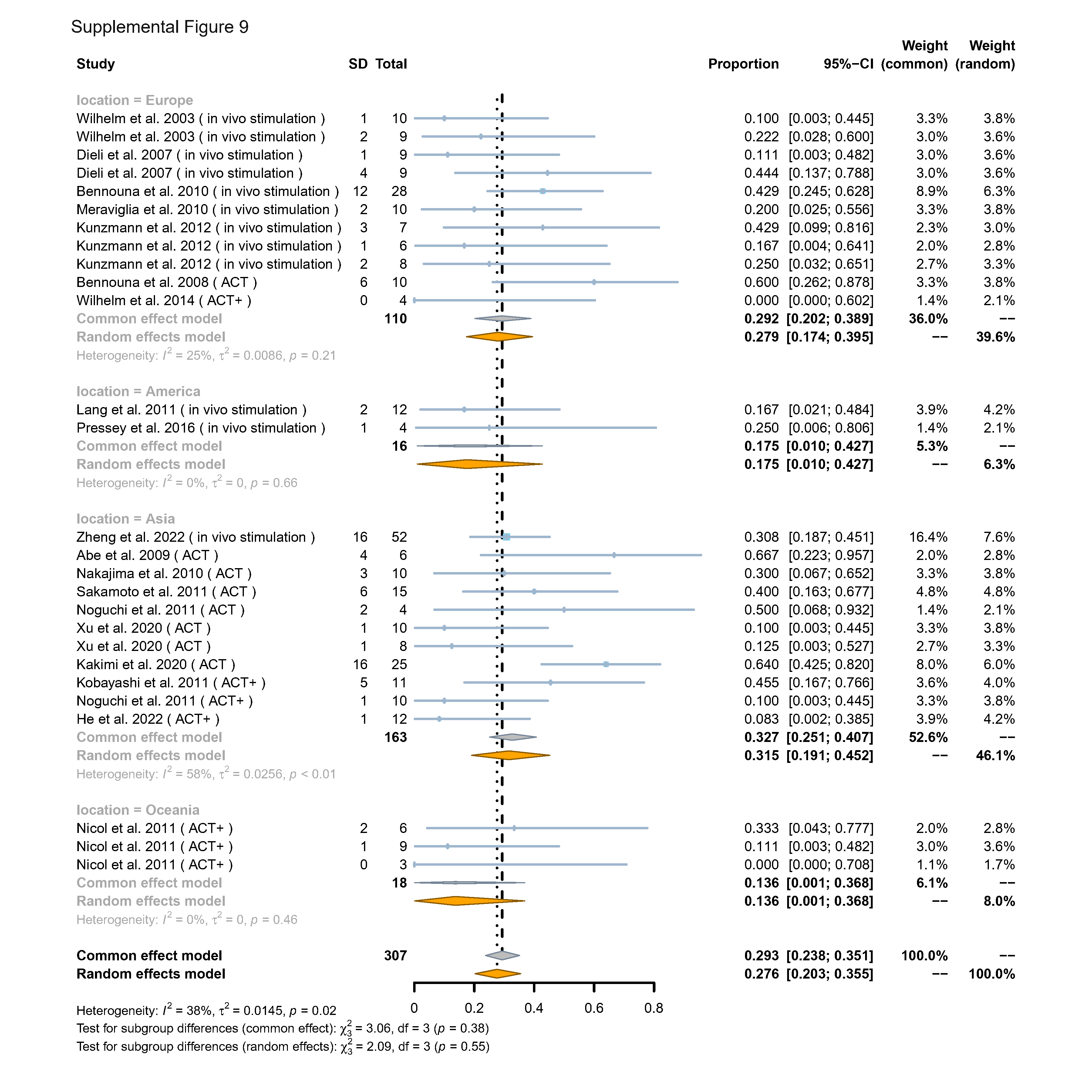

Supplement: Supplementary Figure 9 — Forest plot of stable disease (SD) rate subgrouped by research location (n = 27). [file Image_9.tif]

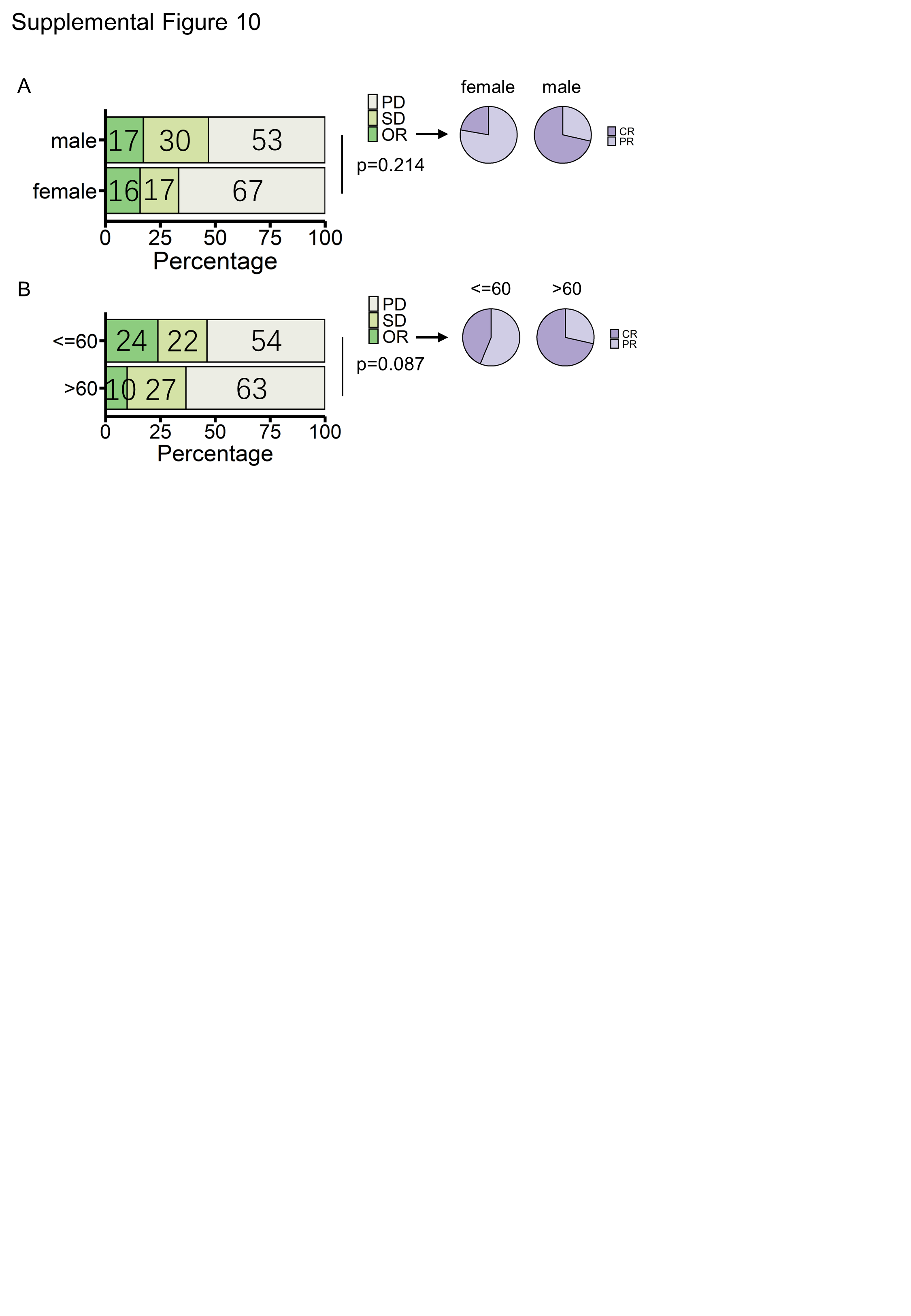

Supplement: Supplementary Figure 10 — Overview of the effects of sex and age influence on outcomes for patients treated with γδ T-cell immunotherapy (n = 138). (A) Proportions of treatment outcomes in male and female patients (male, n = 81; female, n = 57). Pie charts show the proportions of complete response (CR) and partial response (PR) within objective response (OR) patients of each sex (male, n = 14; female, n = 9). (B) Proportions of treatment outcomes in different age groups (≤ 60 years, n = 67; > 60 years, n = 71). Pie charts show the proportions of CR and PR within the OR patients of each age group (≤ 60 years, n = 16; > 60 years, n = 7). p-values (Pearson’s chi-squared test) are indicated in (A, B). To take full advantage of these previous human studies, there were 138 patients for whom detailed information, such as age and sex, was available alongside treatment responses. The overall treatment responses were different among the three strategies (p = 0.000, Pearson’s chi-squared test, n = 138; Table 3), but, in this smaller sample, adoptive cell transfer (ACT) combined with other treatments except for IL-2 treatment (ACT+) had the best response rate [38% OR rate vs. 11% of in vivo stimulation patients (p = 0.013) and 2% of ACT patients (p = 0.000)]. Of these 138 patients, 57 were female and 81 were male. The responses [OR, SD, and progressive disease (PD)] to different treatments were similar when compared between sexes (p = 0.214, Pearson’s chi-squared test, n = 138; Table 3 ), whereas the age distribution was different between female and male patients (p = 0.008, Wilcoxon rank-sum test), with 61% and 40% of female and male patients less than 60 years old, respectively (median age: 57 years for females and 63 years for males). In addition, previous studies indicated that the percentage of Vγ9Vδ2 T cells was lower in elderly people and showed defects in functioning (92, 104); for this reason, the patients here were divided into age groups of ≤ 60 years and > 60 years (in order to have similar [file Image_10.tif]
